# Versatile deprotonated NHC: C,N-bridged dinuclear iridium and rhodium complexes

**DOI:** 10.3762/bjoc.12.13

**Published:** 2016-01-22

**Authors:** Albert Poater

**Affiliations:** 1 Institut de Química Computacional i Catàlisi, Departament de Química, Universitat de Girona, Campus de Montilivi, E-17071 Girona, Spain

**Keywords:** DFT, head-to-head, head-to-tail, iridium, isomerization, N-heterocyclic carbene, rhodium

## Abstract

Bearing the versatility of N-heterocyclic carbene (NHC) ligands, here density functional theory (DFT) calculations unravel the capacity of coordination of a deprotonated NHC ligand (pNHC) to generate a doubly C2,N3-bridged dinuclear complex. Here, in particular the discussion is based on the combination of the deprotonated 1-arylimidazol (aryl = mesityl (Mes)) with [M(cod)(μ-Cl)] (M = Ir, Rh) generated two geometrical isomers of complex [M(cod){µ-C_3_H_2_N_2_(Mes)-κC2,κN3}]_2_). The latter two isomers display conformations head-to-head (H-H) and head-to-tail (H-T) of *C*_S_ and *C*_2_ symmetry, respectively. The isomerization from the H-H to the H-T conformation is feasible, whereas next substitutions of the cod ligand by CO first, and PMe_3_ later confirm the H-T coordination as the thermodynamically preferred. It is envisaged the exchange of the metal, from iridium to rhodium, confirming here the innocence of the nature of the metal for such arrangements of the bridging ligands.

## Introduction

In the framework of organometallic chemistry, N-heterocyclic carbenes (NHC) centre a well stablished class of relatively new ligands since in 1991 Arduengo and collaborators isolated the first stable NHC of the imidazole type with bulky N-substituents [1]. However the existence of stable NHCs was before postulated by Wanzlick et al. during the 1960s [2–4] and supported later by Öfele [5–6]. NHCs have become useful ligands in many transition metal-catalyzed reactions, stimulating the study of the unique features of the M–NHC bond [7–8], which favored the synthesis of new NHCs and to their use as ligands in transition metal complexes. The latter complexes were usually obtained by an easy replacement of a phosphine by the new NHC ligand, displaying a very high stability under many catalytic conditions. Furthermore, NHCs exhibit better activity, despite bearing its carbene functionality. Of course, these good results in basic research supposed and explosion of industrial efforts to design the right metal NHC-based catalyst for any kind of reaction. Anyway, neither a unique nor a few list of catalysts turned out to be effective for any catalytic reaction, but some successful applications were achieved in the ﬁeld of Ru-catalyzed metathesis of oleﬁns [9–12], Ir-catalyzed hydrogenation [13–14], Pd-catalyzed C=C coupling reactions [15–16], Ir-catalyzed CO_2_ fixation [17–18], and/or functionalization of alkenes and alkynes by Au-catalyzed reactions [19–21].

**Scheme 1:**
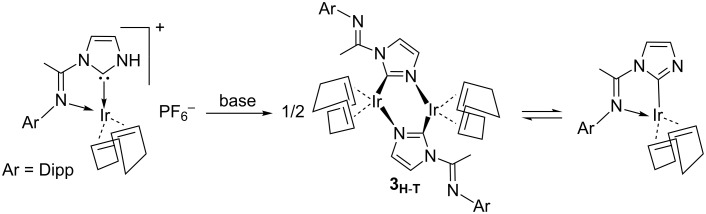
Equilibrium between the monoiridum complex bearing a C-bound anionic imidazolide and its corresponding dimer, once deprotonated a pNHC.

It is not feasible to exclude the asymmetry thanks to the modification of any of the two groups on the imidazolin-2-ylidene ring for two reasons. First, H atoms on the backbone of the either saturated or unsaturated imidazolin-2-ylidene ring suppose a key structural feature for the introduction of asymmetry in the NHC ring. Second, these H atoms might transform the corresponding NHCs in potentially efficient chiral NHCs in asymmetric synthesis [22]. Following with the latter recipe, protic NHCs (pNHCs) consist of the presence of a N-bound H atom. Although most of the studies on NHCs together with metal moieties are based on the interaction of the carbene carbon of the NHC with the metal [23–26], the high instability due the NH group of the pNHCs can induce secondary interactions leading to bifunctional catalysis [27–28], substrate recognition [29] and/or biological systems [30–31]. Among the synthetic methodologies to access to pNHC metal complexes [32–40], recently *N*-arylimine functionalized pNHC iridium complexes were obtained using excess of [Ir(cod)(µ-Cl)]_2_ [41], and next deprotonation of the pNHC leads to an equilibrium between a mononuclear complex containing a C-bound anionic imidazolide [42–44] and its dimer [45–58], where the NHC moiety binds in a µ-C,N bridging mode (see Scheme 1) [44].

**Scheme 2:**
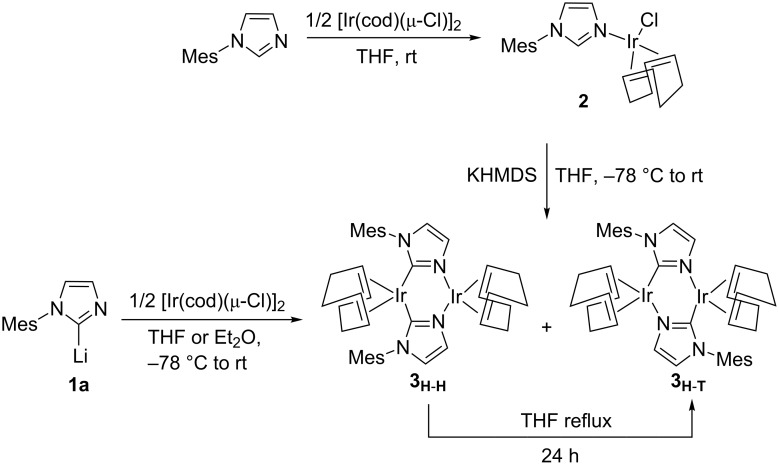
Experimental routes to the “equilibrium” between **3_H_**_-_**_H_** and **3_H_**_-_**_T_**.

Even though during the last two decades thousands of papers have presented and described the NHC based catalysis, bearing a carbene–metal coordination [7], catalytically few efforts have been dedicated to other types of coordination of the NHC with the metal. Braunstein and collaborators have smartly faced the challenge to mix the reactivity of both coordinative atoms of pNHCs [59], either the carbene carbon or the non-substituted nitrogen, i.e., the N from the former N–H group, bearing 1-arylimidazolide ligands. Scheme 2 contains the general scheme that leads to a particular case of the “equilibrium” between two dinuclear complexes, labelled **3_H-H_** and **3_H-T_** (where H-H = head-to-head and H-T = head-to-tail). By DFT calculations here we contribute in the understanding of the thermodynamics of the subsequent C,N-bridged dinuclear iridium and rhodium complexes [59], and the facility for the interconversion between these latter dimeric species.

## Results

**Figure 1:**
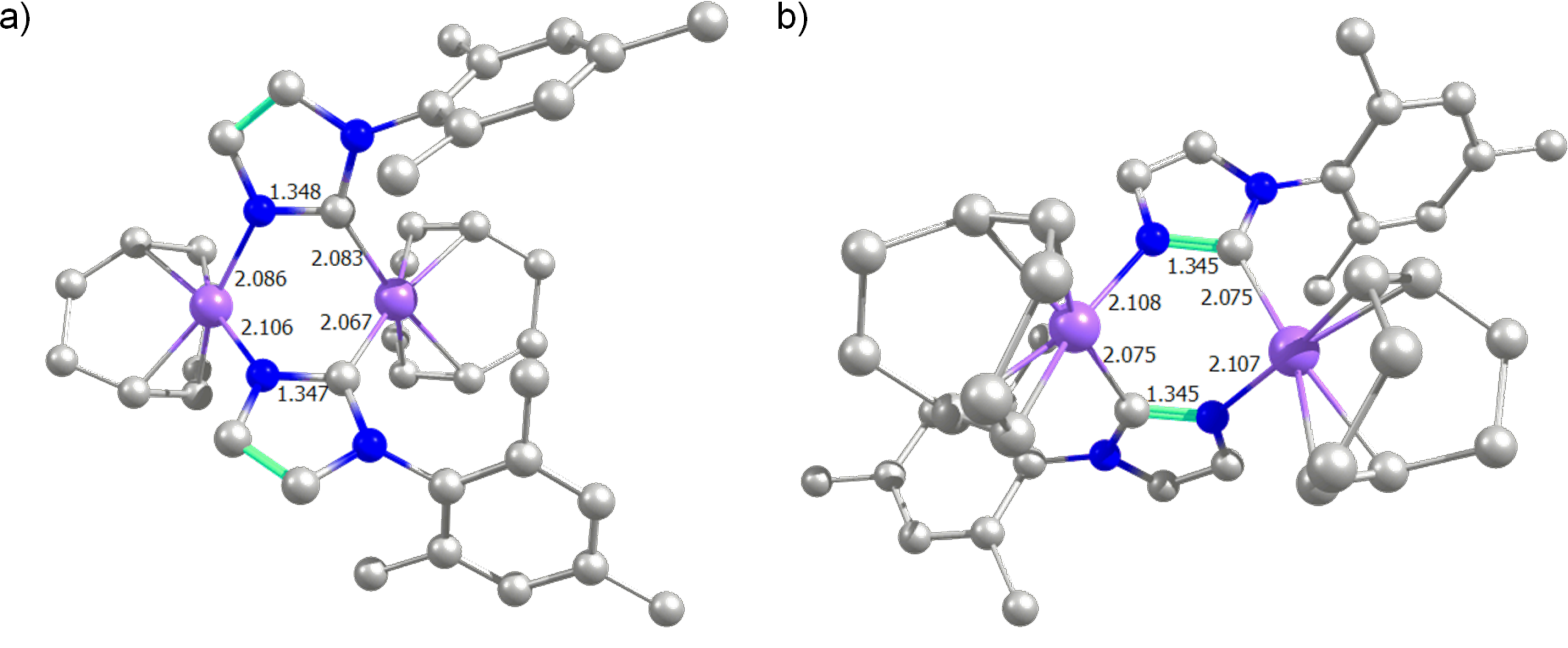
View of the molecular structure of a) **3_H-H_** and b) **3_H-T_**. Hydrogen atoms have been omitted for the sake of clarity (main distances in Å).

To shed light about both isomers of the dinuclear complex **3**, **3_H-H_** and **3_H-T_**, we envisaged DFT calculations (see Figure 1). The optimized geometry of **3_H-H_** is in perfect agreement with the X-ray structure [60] (rmsd = 0.065 Å and 1.1° for the selected main distances and angles) [61–62]. In agreement with experiments that indicated **3_H-T_** is 5.2 kcal/mol more stable than **3_H-H_**, which means that with free tautomerism only the thermodynamic isomer **3_H-T_** would not coexist with **3_H-H_**, but as the unique isomer [59].

Furthermore, the strength of the two H-H and H-T arrangements of the bridging pNHC ligand with the iridium was examined with the Mayer bond order (MBO) [63], which is valuable for evaluating bonding in main group compounds, but has been also used as a tool for the characterization of transition metal systems [64–65]. For **3_H-H_** the NHC coordination to the metal through the carbene carbon, the MBO is 0.874, whereas through the N atom is only 0.438. The type of coordination of pNHCs around the iridium atoms, either H-H or H-T, does not modify significantly the strength of the Ir–C and Ir–N bonds. For **3_H-T_** the MBO values are 0.841 and 0.410. Further, the structure of the dinuclear complexes may be determined by steric effects of both monomeric moieties. To evaluate only the sterics, topographic steric maps were used, which are calculated through the SambVca package developed by Cavallo et al. [66]. This analysis allows the rationalization of the ﬁrst coordination sphere around metal centres where catalytic processes take place. Basically the method calculates the buried volume of a given ligand [67] based on the quantification of the proportion of the ﬁrst coordination sphere of the metal occupied by this ligand. The encumbered zones (color-coded in brown) belong to the part of the ligand that protrudes in the direction of the reacting groups, thus restricting the space they can fill, whereas empty zones (color coded in blue) correspond to the part where the ligand retracts from the reacting groups [68–69].

**Figure 2:**
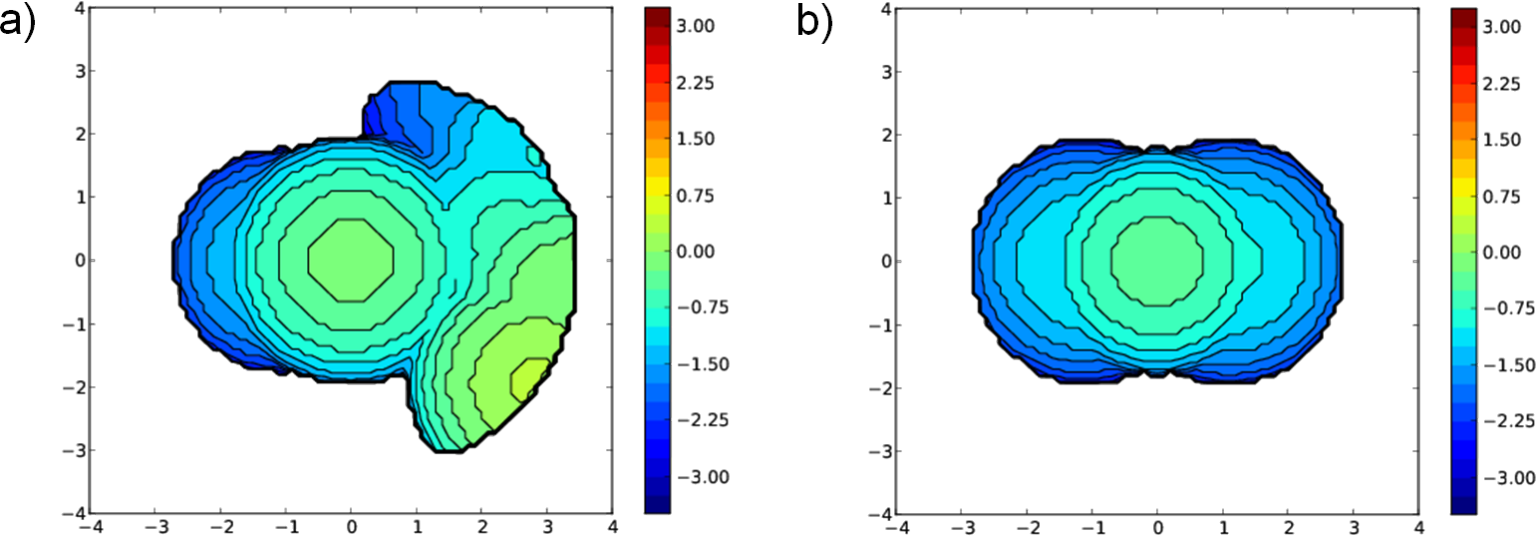
Steric maps for the NHC ligand of **3_H_**_-_**_H_**, coordinated to iridium by a) carbene or b) nitrogen. The isocontour curves are given in Å. The systems are oriented along the *z*-axis defined by the metal and the coordinating atom bonded to the metal. The two maps are computed with a radius of the sphere equal to 3.5 Å.

For **3_H-H_** the percentage of buried volume (%VBur) is 26.2 bearing a metal–carbene coordination, whereas it decreases to only 19.1 when the NHC bonds to the metal through the deprotonated nitrogen atom. However, the steric maps in Figure 2 confirm completely that the system prefers the latter N-bound coordination. Splitting the map into four quadrants the carbene coordination reveals a quadrant highly sterically hindered (37.2) due to the rotation of the aromatic ring on the NHC which facilitates the allocation of such a NHC ligand in the dinuclear complexes. The other quadrant where this aromatic ring participates displays a value of 29.2, whereas the other two are innocuous for the metal sphere with low values of 19.3 and 19.1. On the other hand, the coordination of the NHC through the nitrogen is sterically innocent towards the metal sphere because the four quadrants show low sterical occupations (19.1, 18.7, 19.4 and 19.1). Thus, the N-bound coordination of the NHC can be regarded as the perfect coordination to facilitate a free “ﬁrst coordination sphere”, with enough space for allowing the formation of a dinuclear complex. For **3_H-T_** the %VBur is nearly identical, displaying values of 26.4 and 19.0, for carbene and nitrogen coordination to the iridium centre, respectively.

Natural bond order (NBO) analysis on the iridium reveal that the two equivalent iridium centres in **3_H-T_** display a charge on the metal of 0.009*e*, whereas −0.143 and 0.189*e* for **3_H-H_** for the iridium bonded to two carbene carbons and two nitrogen atoms of the NHC, respectively. This confirms that the metal–carbene coordination allocates more electron density on the metal than through the nitrogen.

Despite the dimeric nature of complex **3**, hypothetically they might be discussed as aggregates of two monomeric moieties. However the coupling between the two metal centres seems demonstrated by removing an electron of the system, thus the expected mixed valent Ir(I)/Ir(II) species turns out to display two identical metal centres that distribute equally the cost of the electrolysis of **3**. Geometrically no asymmetry is observed, and together with the positive charge increase of 0.306*e* on each former Ir(I) centre, shows that the effect on system **3** of the released electron is mainly paid by the metal centres, but also partially spread over the ligands [59].

**Scheme 3:**
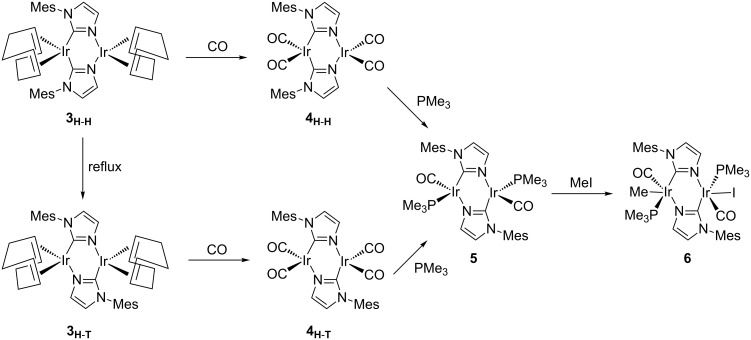
Equilibrium between complexes **3**–**6**, in the presence of CO, PMe_3_, and MeI.

To follow up the experimental results the tautomerism/metallotropism between pNHC and imidazole ligands in these iridium complexes bearing a doubly C,N-bridged dinuclear core was also computationally studied after the displacement of cod ligands of **3_H-H_** and **3_H-T_** by CO in Scheme 3 [59], affording the tetracarbonyl complexes [M(CO)_2_{µ-C_3_H_2_N_2_(Mes)-κC2,κN3}]_2_, **4_H-H_** and **4_H-T_**, respectively. Next we evaluated the substitution of CO ligands by PMe_3_ affording complex [M(CO)(PMe_3_){µ-C_3_H_2_N_2_(Mes)-κC2,κN3}]_2_ (**5**) and finally, oxidative addition of MeI to the latter complex **5** afforded the dinuclear complex [M(CO)_2_(PMe_3_)_2_(Me)I{µ-C_3_H_2_N_2_(Mes)-κC2,κN3}]_2_.

**Figure 3:**
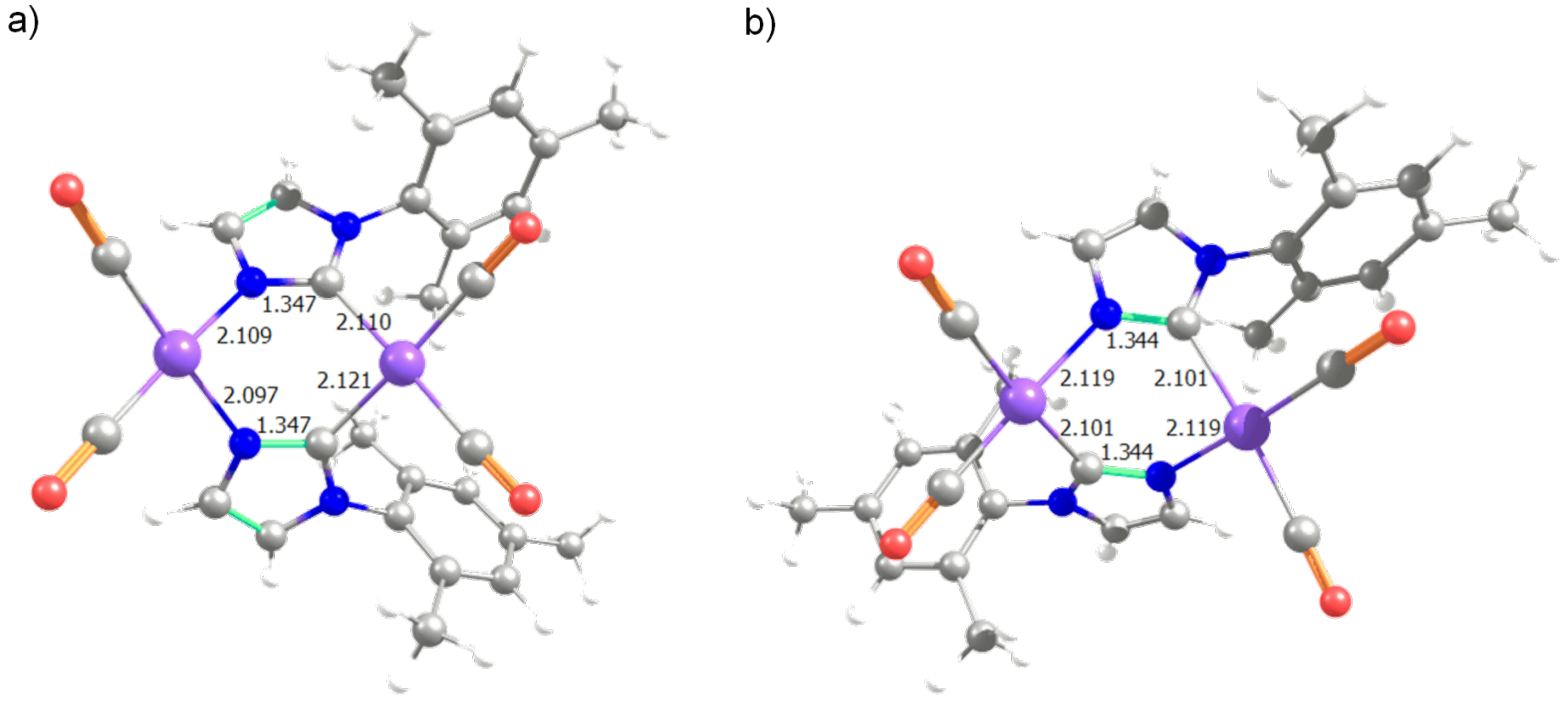
View of the molecular structure of a) **4_H_**_-_**_H_** and b) **4_H_**_-_**_T_** (main distances in Å).

In agreement with experiments [59], without reflux conditions, the substitution of cod ligands by CO is extremely favored, being isomer **4_H-T_** 48.5 kcal/mol more stable than **3_H-T_**, and again the equilibrium between **4_H-H_** and **4_H-T_** is displaced towards the latter species, by a difference of 3.0 kcal/mol. Furthermore, the third CO ligand coordination on each iridium atom in **4** was faced but discarded due to a destabilization of 6.7 and 14.8 kcal/mol with respect to **4_H-H_** and **4_H-T_**, respectively. Basically this lower stability is not only due to the sterical hindrance, but to the preferred quasi perfect square planar type of coordination on each iridium centre (see Figure 3). Going into electronic details, this **3**→**4** transformation also follows the principle of maximum hardness [70–71], i.e., the chemical hardness evolves from 39.6 to 46.7 kcal/mol bearing a H-T type of coordination. This increase of chemical hardness is a consequence of the increased stability of the HOMO, which results in a larger HOMO–LUMO gap [61,65]. To point out that the two types of coordination, H-H and H-T, do not suppose a significant change of chemical hardness, just an increase for H-T of only 0.6 kcal/mol for species **4**, whereas a decrease of 0.1 for species **3**. Thus the electronics do not affect the equilibrium between both arrangements of the bridging ligands, but sterics as stated above. Further, the NBO charges show a decrease of the charge on both metals of 0.777*e* in the **3**→**4** transformation. Despite the π-backdonation of CO the donation to the iridium centres is larger than the corresponding electron density transferred by the cod ligands in species **3**.

**Figure 4:**
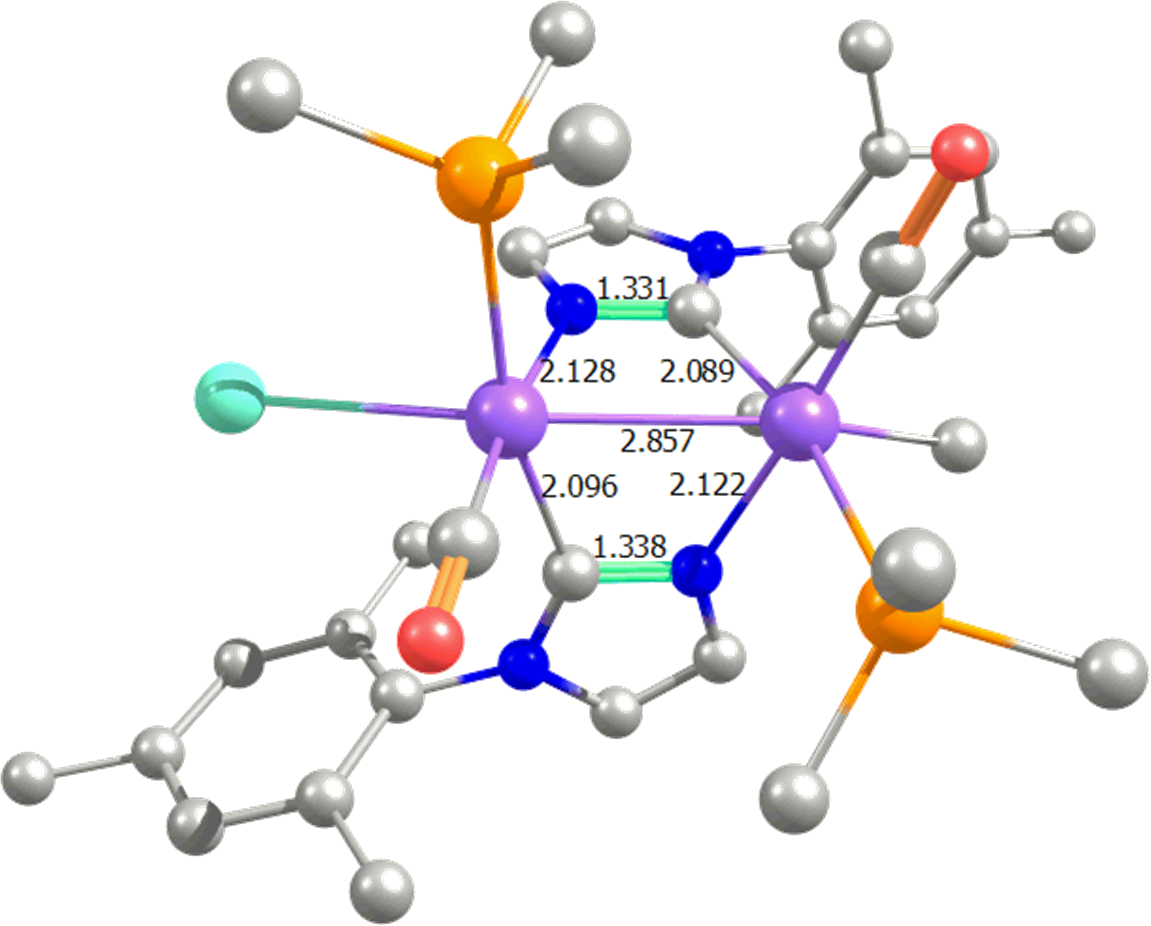
View of the molecular structure of **6**. Hydrogen atoms have been omitted for the sake of clarity (main distances in Å).

**4_H-H_** and **4_H-T_** evolves to complex **5**, exchanging one CO by a PMe_3_ ligand on each iridium centre, releasing 5.0 and 2.0 kcal/mol, respectively. Bearing an energy difference of 11.7 kcal/mol the H-T isomer is favored with respect to the H-H, in agreement with experiments [59], because the H-H coordination was not locate for **5**, and further thermodynamics do not support the H-H coordination for **5**, with an energy endergonicity of 6.7 kcal/mol with respect the previous corresponding complex **4**. Oxidative addition of MeI to **5** affords complex **6**, in an exergonic release of 16.5 kcal/mol, pointing out that there are several other isomers of complex **6** that differ from the Me and I coordination to each corresponding iridium centre. However all these alternative isomers of complex **6** are placed higher in energy by at least 12.9 kcal/mol (see Supporting Information File 1). The Ir–Ir distance in **6** is only 2.857 Å (see Figure 4), which is a clear proof of concept of a formally metal–metal bonded d^7^–d^7^ complex, to be compared with complex **5**, where this Ir–Ir distance elongates till 3.497 Å. Furthermore, the MBO for the metal–metal bond reveals a significant value of 0.626 for complex **6** [72], being null for **5** and the previous complexes. Electronically, the charge on metals for complex **6** is 0.334 less charged, which is explained by, among other reasons, the weaker back-bonding from the metal to the CO ligands compared to **5**, which affords the metal–metal interaction in **6**.

To extent the scope of the reactivity of the NHC here, the cod ligands were first exchanged by ethylene, revealing a preferred H-T type of coordination of the NHC (5.1 kcal/mol). On the other hand, the iridium was replaced by rhodium to verify the innocent nature of the electronic properties of the metal, maintaining nearly completely the geometry features for complexes **3**–**6**. Bearing rhodium complex **3_H-T_** is again more stable than **3_H-H_**, even 0.2 kcal/mol more stable. To sum up, no change of behaviour between iridium and homologous rhodium complexes was observed. On the other hand, the evolution from H-T to H-H arrangement was evaluated and mechanistically this process is predicted to be dissociative since neither intramolecular transition state was located nor the linear transits suggested energy barriers affordable at the experimental conditions.

## Conclusion

The two possible H-T and H-H arrangements of a bridging NHC here have been studied in detail by DFT calculations. This is a contribution in the understanding of the thermodynamics of the subsequent C,N-bridged dinuclear iridium and rhodium complexes defined by Braunstein et al. [59] and the facility for the interconversion between these latter dimeric species. Steric maps confirm that the H-T is preferred since the metal centres are less sterically hindered, thus revealing the relatively unstable kinetic H-H isomers with respect to the H-T ones. However, screening the evolution from complex **3** to **6**, going through complexes **4** and **5**, the relative thermodynamics show a constant and sharp decay of energy, not due to steric factors, but mainly electronics.

## Computational Details

The density functional calculations were performed on all the systems with the Gaussian 09 set of programs [73], Revision D.01, at the BP86 GGA level [74–76], adding the Grimme D3 dispersion term [77]. For iridium and rhodium we used the small-core, quasi-relativistic Stuttgart/Dresden (SDD) effective core potential with an associated valence contracted basis set (standard SDD keywords in Gaussian 09) [78–80]. The electronic configuration of the molecular systems was described with the triple-ζ valence plus polarization (TZVP keyword in Gaussian) basis set on all main group atoms during geometry optimizations [81]. The reported energies have been obtained via single point calculations on the BP86 geometries with triple-ζ valence plus polarization using the M06 functional [82]. Solvent effects, using either tetrahydrofuran, dichloromethane or toluene, were calculated with the polarizable continuous solvation model polarizable continuum model (PCM) model [83–84], and non-electrostatic terms were also included. The cavity is created via a series of overlapping spheres. However, the numbers reported throughout the text here are based on THF because this is the solvent employed in the experiments bearing the H-H to H-T rearrangement [59]. The geometry optimizations were performed without symmetry constraints, and the nature of the extrema was checked by analytical frequency calculations. Furthermore, all the extrema were confirmed by calculation of the intrinsic reaction paths.

The reported free energies in this work include energies obtained at the M06/TZVP level of theory in solvent corrected with zero-point energies at 298.15 K, together with the model of Martin et al. [85] which consists of thermal corrections and entropy effects evaluated at 1354 atm [86–89], with the BP86-d3/TZVP method in the gas phase [90–91].

%VBur calculations: The buried volume calculations were performed with the SambVca package developed by Cavallo et al. [90]. The radius of the sphere around the metal centre was set to 3.5 Å, while for the atoms we adopted the Bondi radii scaled by 1.17, and a mesh of 0.1 Å was used to scan the sphere for buried voxels. The steric maps were evaluated with a development version of the SambVca package.

## Supporting Information

File 1 Energies, cartesian coordinates, and 3D view for all DFT optimized species.
