# Supplementary material for: Versatile deprotonated NHC: C,N-bridged dinuclear iridium and rhodium complexes
Source: Beilstein J Org Chem. 2016 Jan 22;12:117–24. doi: 10.3762/bjoc.12.13 (PMC4734348; doi:10.3762/bjoc.12.13)
Supplement: File 1 — Energies, cartesian coordinates, and 3D view for all DFT optimized species. [file Beilstein_J_Org_Chem-12-117-s001.pdf]

Supporting Information

for

Versatile deprotonated NHC: C,N-bridged dinuclear iridium and rhodium complexes

Albert Poater*

Address: Institut de Química Computacional i Catàlisi, Departament de Química, Universitat de Girona, Campus de Montilivi, E-17071 Girona, Spain

Email: Albert Poater - albert.poater@udg.edu

*Corresponding author

Energies, cartesian coordinates, and 3D view for all DFT optimized species

xyz coordinates and absolute energies (in a.u.)

$3_{H\text{-}H}$

Zero-point correction= 0.817131 (Hartree/Particle)
Thermal correction to Energy= 0.862343
Thermal correction to Enthalpy= 0.863287
Thermal correction to Gibbs Free Energy= 0.739440
Sum of electronic and zero-point Energies= -1982.147291
Sum of electronic and thermal Energies= -1982.102079
Sum of electronic and thermal Enthalpies= -1982.101135
Sum of electronic and thermal Free Energies= -1982.224982

C	-0.035335	1.117004	-0.985033
C	-1.290112	1.642900	-2.756341
H	-2.121213	1.565469	-3.434966
C	-0.196670	2.442029	-2.815817
H	0.118393	3.185728	-3.525713
C	1.621589	3.000172	-1.233934
C	1.243583	4.256104	-0.741631
C	2.239350	5.114909	-0.273940
H	1.950279	6.083418	0.119628
C	3.581011	4.749075	-0.270485
C	3.921139	3.485326	-0.754539
H	4.960898	3.175846	-0.746618
C	2.964262	2.599433	-1.240346
C	-0.203574	4.666671	-0.664501
H	-0.560355	5.067566	-1.617287
H	-0.344304	5.436797	0.095190
H	-0.845888	3.820285	-0.418703
C	4.646998	5.677986	0.248479
H	4.221873	6.614116	0.614338
H	5.370690	5.923507	-0.534248
H	5.204611	5.219602	1.070149
C	3.355323	1.237321	-1.720925
H	3.009833	1.069677	-2.742979
H	2.895909	0.471756	-1.092674
H	4.436933	1.101368	-1.692368
C	-0.110893	1.636679	2.132225
H	-0.804161	2.228043	1.547701
C	1.256017	1.797200	1.922180
H	1.586646	2.513470	1.188812
C	2.294953	1.395907	2.949491
H	3.207821	1.961502	2.755268
H	1.956511	1.705213	3.944697
C	2.641784	-0.120496	2.983772
H	2.708072	-0.443579	4.031471
H	3.635368	-0.272758	2.559085
C	1.676594	-1.026106	2.248876
H	2.156111	-1.809498	1.684516
C	0.331234	-1.172074	2.580449
H	-0.178933	-2.073393	2.262154
C	-0.347943	-0.419619	3.709055
H	-1.274080	-0.942113	3.954795
H	0.275711	-0.478546	4.608957
C	-0.676651	1.077900	3.422546
H	-0.295491	1.681076	4.255536
H	-1.757369	1.220971	3.419230
C	0.083183	-1.675497	-0.412268
C	-1.335634	-3.150360	-1.316011
H	-2.276362	-3.510871	-1.692374
C	-0.130492	-3.766991	-1.267928
H	0.205202	-4.732872	-1.600795
C	2.162384	-3.067008	-0.680281
C	2.773187	-3.768829	0.363425
C	4.159834	-3.924438	0.323845
H	4.647663	-4.454648	1.135719
C	4.928231	-3.425462	-0.724894
C	4.275138	-2.791217	-1.781959
H	4.852595	-2.431229	-2.626366
C	2.893708	-2.620423	-1.789475
C	1.988073	-4.372752	1.500635
H	2.303372	-3.960184	2.461296
H	2.152034	-5.452998	1.539938
H	0.919584	-4.194812	1.398139
C	6.425648	-3.593887	-0.722221
H	6.881096	-3.146293	-1.607035
H	6.705069	-4.651209	-0.700683
H	6.873307	-3.124032	0.158125
C	2.180648	-2.046811	-2.984290
H	2.890276	-1.680449	-3.725758
H	1.507864	-1.234740	-2.708983
H	1.563778	-2.815769	-3.457940
C	-3.547237	-1.313423	1.087116
H	-2.809106	-2.005292	1.477705
C	-4.226627	-1.657174	-0.098114
H	-4.017460	-2.620663	-0.548646
C	-5.578380	-1.051626	-0.458822
H	-6.139717	-1.766772	-1.063728
H	-6.166872	-0.912353	0.453806
C	-5.491698	0.296072	-1.222427
H	-6.324267	0.939225	-0.905404
H	-5.634255	0.122139	-2.290443
C	-4.176098	1.031625	-1.039590
H	-3.872465	1.620939	-1.897907
C	-3.648312	1.383809	0.217416

H	-2.930789	2.196532	0.249563
C	-4.359841	1.091395	1.530839
H	-5.437040	1.231906	1.393300
H	-4.056457	1.836923	2.268671
C	-4.102223	-0.329544	2.101494
H	-3.410909	-0.276828	2.943198
H	-5.043446	-0.720481	2.509710
N	-1.185025	0.845945	-1.633453
N	0.580006	2.130316	-1.702146
N	-1.190763	-1.878053	-0.804786
N	0.748470	-2.853597	-0.693395
Ir	0.483106	0.039067	0.700692
Ir	-2.569889	-0.330335	-0.568601

$3_{H\text{-}T}$

Zero-point correction= 0.816113 (Hartree/Particle)
Thermal correction to Energy= 0.861937
Thermal correction to Enthalpy= 0.862881
Thermal correction to Gibbs Free Energy= 0.736114
Sum of electronic and zero-point Energies= -1982.154705
Sum of electronic and thermal Energies= -1982.108882
Sum of electronic and thermal Enthalpies= -1982.107937
Sum of electronic and thermal Free Energies= -1982.234704

C	-1.324542	-0.587436	0.888787
C	-0.302253	-1.436427	2.677464
H	0.511102	-1.619347	3.357418
C	-1.573195	-1.914449	2.700187
H	-2.078005	-2.588648	3.368708
C	-3.554325	-1.680000	1.212604
C	-4.605538	-1.095391	1.926181
C	-5.911085	-1.392670	1.538634
H	-6.734494	-0.937892	2.078433
C	-6.181133	-2.242344	0.468079
C	-5.107045	-2.813366	-0.213052
H	-5.301772	-3.485236	-1.042235
C	-3.785343	-2.550815	0.141616
C	-4.331747	-0.132916	3.052492
H	-5.245369	0.376279	3.358609
H	-3.599968	0.619407	2.751424
H	-3.918006	-0.642020	3.925744
C	-7.598512	-2.514972	0.032616
H	-8.308597	-2.322454	0.838159
H	-7.723566	-3.550345	-0.290193
H	-7.875066	-1.875019	-0.810496
C	-2.642335	-3.177707	-0.609401
H	-2.993457	-3.993972	-1.241155
H	-1.883861	-3.565456	0.073153
H	-2.144967	-2.437585	-1.238587
C	-2.522644	2.200544	-0.040229
H	-2.351161	2.226809	1.031969
C	-3.436339	1.227554	-0.504697
H	-3.926981	0.617508	0.241935
C	-4.211410	1.293313	-1.804201
H	-5.085434	1.949629	-1.697345
H	-4.601208	0.289454	-1.991444
C	-3.355277	1.734265	-3.009717
H	-3.384624	2.819559	-3.119338
H	-3.788636	1.328152	-3.926183
C	-1.909401	1.273407	-2.894409
H	-1.613677	0.496544	-3.592929
C	-0.883459	2.048195	-2.341688
H	0.130808	1.814582	-2.652513
C	-1.042981	3.420719	-1.726283
H	-1.121897	4.188320	-2.507120
H	-0.122680	3.630842	-1.178584
C	-2.239124	3.518539	-0.756324
H	-2.035248	4.292635	-0.013116
H	-3.134687	3.844407	-1.288519
C	1.322797	-0.604997	-0.871863
C	1.572878	-1.972021	-2.653599
H	2.077803	-2.662197	-3.305428
C	0.302179	-1.493497	-2.642910
H	-0.511462	-1.693695	-3.317676
C	3.557443	-1.695470	-1.181127
C	4.600179	-1.150939	-1.936955
C	5.910875	-1.434817	-1.553971
H	6.727561	-1.008831	-2.126258
C	6.193541	-2.231181	-0.447287
C	5.126728	-2.757406	0.280912
H	5.331137	-3.382208	1.144107
C	3.801512	-2.508399	-0.067546
C	4.316223	-0.241024	-3.103894
H	5.219205	0.285039	-3.413232
H	3.555594	0.497758	-2.844671
H	3.936519	-0.795765	-3.965070
C	7.614261	-2.523251	-0.035791
H	8.330905	-2.032501	-0.695260
H	7.817472	-3.597014	-0.059378
H	7.806234	-2.179567	0.983778
C	2.670432	-3.079239	0.743985
H	2.240807	-2.310869	1.390173
H	3.017365	-3.904860	1.366043
H	1.860198	-3.436150	0.106699
C	2.520097	2.205395	0.001860

H	2.349587	2.211548	-1.070797
C	3.433611	1.241413	0.484714
H	3.925209	0.618984	-0.250950
C	4.206349	1.327670	1.784280
H	5.084669	1.976346	1.667197
H	4.589163	0.324862	1.990825
C	3.350414	1.795132	2.979634
H	3.782718	1.407346	3.904533
H	3.381214	2.882403	3.066908
C	1.904331	1.333000	2.872438
H	1.608031	0.570222	3.586121
C	0.878541	2.096652	2.304191
H	-0.135670	1.868595	2.618981
C	1.036870	3.456539	1.661319
H	1.111853	4.240484	2.426199
H	0.117593	3.652850	1.106811
C	2.234905	3.536634	0.692522
H	2.032280	4.296376	-0.065694
H	3.129181	3.873006	1.220191
Ir	-1.433210	0.526156	-0.858972
Ir	1.429890	0.546216	0.851733
N	-0.162010	-0.636042	1.563357
N	-2.208740	-1.375264	1.588147
N	2.208016	-1.407158	-1.554267
N	0.160320	-0.670163	-1.545794

$4_{H\text{-}H}$

Zero-point correction= 0.485143 (Hartree/Particle)
Thermal correction to Energy= 0.524545
Thermal correction to Enthalpy= 0.525489
Thermal correction to Gibbs Free Energy= 0.408785
Sum of electronic and zero-point Energies= -1811.708907
Sum of electronic and thermal Energies= -1811.669505
Sum of electronic and thermal Enthalpies= -1811.668561
Sum of electronic and thermal Free Energies= -1811.785265

C	-0.370303	1.295609	-0.515391
C	-1.796647	2.188976	-1.976882
H	-2.718314	2.287430	-2.522581
C	-0.655644	2.915119	-2.059894
H	-0.380994	3.755431	-2.671868
C	1.466643	2.987544	-0.775321
C	1.413155	4.158679	-0.007333
C	2.613644	4.771725	0.338260
H	2.586037	5.671495	0.942802
C	3.845218	4.247151	-0.049933
C	3.857297	3.078819	-0.807244
H	4.806619	2.654835	-1.115908
C	2.681290	2.430569	-1.182246
C	0.100144	4.716031	0.476695
H	-0.480883	5.150923	-0.339487
H	0.264315	5.489828	1.225658
H	-0.517937	3.934702	0.923395
C	5.130763	4.905447	0.379953
H	5.031101	5.991840	0.413305
H	5.951313	4.659209	-0.295659
H	5.417098	4.572438	1.381923
C	2.727959	1.149683	-1.962960
H	1.963333	1.124282	-2.739558
H	2.540295	0.302824	-1.298669
H	3.703768	1.009666	-2.428741
C	1.171430	1.447150	1.919369
C	1.205500	-1.233128	2.177366
C	-0.448984	-1.573313	-0.192356
C	-2.036542	-2.793099	-1.178121
H	-3.024123	-3.024522	-1.535727
C	-0.891534	-3.513087	-1.262040
H	-0.672627	-4.470195	-1.700357
C	1.473651	-3.109913	-0.607661
C	1.955492	-3.920842	0.421032
C	3.322645	-4.200527	0.443245
H	3.715930	-4.810792	1.248928
C	4.188435	-3.715146	-0.532326
C	3.656001	-2.959060	-1.578139
H	4.312075	-2.602929	-2.364958
C	2.300344	-2.655048	-1.643176
C	1.039943	-4.521426	1.455946
H	1.551224	-4.624422	2.412414
H	0.714346	-5.516465	1.138857
H	0.145673	-3.921269	1.611949
C	5.669944	-3.979871	-0.453726
H	6.112354	-4.071955	-1.447345
H	5.884117	-4.894750	0.100679
H	6.180928	-3.158493	0.057367
C	1.721497	-1.920849	-2.823729
H	2.507387	-1.457594	-3.418887
H	1.018852	-1.147721	-2.517067
H	1.173714	-2.613410	-3.468761
C	-4.358165	-1.208732	0.561412
C	-4.237948	1.439125	0.050242
N	-1.613046	1.202495	-1.026859
N	0.223778	2.364945	-1.140291
N	-1.752146	-1.606006	-0.530333
N	0.086414	-2.752572	-0.643916
Ir	0.469712	-0.012055	0.911129

Ir	-3.097936	-0.035199	-0.182939
O	-4.921017	2.350005	0.181250
O	-5.117088	-1.939906	1.011927
O	1.658112	-1.914403	2.980458
O	1.567650	2.294382	2.581020

$4_{H\text{-}T}$

Zero-point correction= 0.484522 (Hartree/Particle)
Thermal correction to Energy= 0.524314
Thermal correction to Enthalpy= 0.525259
Thermal correction to Gibbs Free Energy= 0.407200
Sum of electronic and zero-point Energies= -1811.713254
Sum of electronic and thermal Energies= -1811.673461
Sum of electronic and thermal Enthalpies= -1811.672517
Sum of electronic and thermal Free Energies= -1811.790575

C	-1.363522	0.791904	0.127423
C	-0.473268	2.663656	0.928963
H	0.285380	3.410697	1.082411
C	-1.737519	2.597742	1.418202
H	-2.291150	3.246077	2.073296
C	-3.602833	0.953321	1.211403
C	-4.697958	1.527277	0.558526
C	-5.966784	1.038600	0.860427
H	-6.825737	1.465286	0.354354
C	-6.157320	0.010971	1.782619
C	-5.040484	-0.525462	2.420947
H	-5.174196	-1.320053	3.146837
C	-3.751035	-0.070574	2.152782
C	-4.512874	2.631199	-0.449175
H	-5.424278	2.783633	-1.026144
H	-3.703905	2.403983	-1.144112
H	-4.258721	3.575679	0.038796
C	-7.537228	-0.531022	2.054264
H	-8.293841	0.252946	1.990525
H	-7.600542	-0.985740	3.044064
H	-7.800210	-1.299552	1.321418
C	-2.557671	-0.677218	2.841182
H	-2.864673	-1.251428	3.715371
H	-1.845621	0.086339	3.158965
H	-2.023017	-1.344569	2.161620
C	-3.134765	-0.526479	-1.691476
C	-1.563081	-2.768690	-1.719189
C	1.363555	-0.791968	0.127440
C	1.737539	-2.597545	1.418593
H	2.291172	-3.245763	2.073803
C	0.473273	-2.663526	0.929409
H	-0.285390	-3.410517	1.083005
C	3.602857	-0.953181	1.211534
C	4.698054	-1.527361	0.558954
C	5.966842	-1.038558	0.860767
H	6.825845	-1.465428	0.354934
C	6.157287	-0.010572	1.782593
C	5.040402	0.526032	2.420674
H	5.174045	1.320866	3.146312
C	3.750976	0.071002	2.152602
C	4.512991	-2.631563	-0.448441
H	5.424961	-2.785365	-1.024150
H	3.705207	-2.403659	-1.144543
H	4.257018	-3.575505	0.039603
C	7.537152	0.531607	2.054080
H	8.293882	-0.252230	1.990177
H	7.600540	0.986274	3.043899
H	7.799898	1.300224	1.321238
C	2.557581	0.677822	2.840795
H	2.023264	1.345411	2.161205
H	2.864505	1.251820	3.715153
H	1.845264	-0.085608	3.158275
C	3.134828	0.526208	-1.691464
C	1.563092	2.768497	-1.719466
Ir	-1.540590	-1.049330	-0.868963
Ir	1.540562	1.049161	-0.869191
N	-0.252863	1.548609	0.143134
N	-2.285694	1.432715	0.908879
N	2.285756	-1.432662	0.908990
N	0.252893	-1.548632	0.143349
O	4.110198	0.203032	-2.202707
O	1.563649	3.799482	-2.215923
O	-1.563638	-3.799605	-2.215785
O	-4.110048	-0.203368	-2.202921

5

Zero-point correction= 0.696680 (Hartree/Particle)
Thermal correction to Energy= 0.747773
Thermal correction to Enthalpy= 0.748717
Thermal correction to Gibbs Free Energy= 0.607409
Sum of electronic and zero-point Energies= -2507.144255
Sum of electronic and thermal Energies= -2507.093163
Sum of electronic and thermal Enthalpies= -2507.092218
Sum of electronic and thermal Free Energies= -2507.233527

C	1.369143	-0.812140	0.574257
C	0.418133	-2.609072	1.469161
H	-0.374333	-3.304245	1.684825
C	1.699777	-2.587217	1.922320
H	2.239274	-3.232019	2.593019
C	3.629099	-1.026439	1.586113
C	4.674490	-1.640056	0.889895
C	5.971429	-1.191912	1.128189
H	6.792800	-1.653979	0.591371
C	6.236076	-0.157229	2.023955
C	5.166594	0.430087	2.697256
H	5.357513	1.235536	3.398294
C	3.852408	0.013191	2.493566
C	4.398958	-2.726691	-0.115537
H	5.325307	-3.082723	-0.565637
H	3.756415	-2.354126	-0.915218
H	3.887890	-3.577769	0.339214
C	7.644985	0.339188	2.228157
H	8.368397	-0.476001	2.166093
H	7.761419	0.823592	3.199035
H	7.911876	1.071616	1.460388
C	2.704144	0.676906	3.203843
H	3.062123	1.324183	4.004837
H	2.018221	-0.057823	3.629680
H	2.123036	1.278073	2.500755
C	2.985174	0.438952	-1.440870
C	-1.370485	0.812545	0.569515
C	-1.703232	2.591130	1.912468
H	-2.243817	3.237561	2.580715
C	-0.420961	2.611983	1.461062
H	0.371209	3.307672	1.676128
C	-3.632367	1.030615	1.577511
C	-4.675052	1.639625	0.874009
C	-5.972771	1.189626	1.107266
H	-6.791421	1.643973	0.559988
C	-6.240342	0.160608	2.007956
C	-5.172168	-0.426849	2.684160
H	-5.364548	-1.235959	3.380711
C	-3.858120	-0.008751	2.485408
C	-4.396129	2.719498	-0.137765
H	-5.320614	3.069997	-0.595951
H	-3.747517	2.343592	-0.930859
H	-3.890409	3.575077	0.314593
C	-7.656056	-0.289869	2.264787
H	-8.287078	-0.139524	1.387153
H	-8.101013	0.275030	3.089672
H	-7.694973	-1.346855	2.534015
C	-2.711288	-0.674649	3.195987
H	-2.131321	-1.277546	2.493441
H	-3.070702	-1.320402	3.997570
H	-2.023898	0.059030	3.621245
C	-2.980526	-0.443467	-1.448369
Ir	1.466031	0.930430	-0.534809
Ir	-1.464763	-0.932828	-0.535412
N	0.228811	-1.519861	0.641543
N	2.286728	-1.465480	1.352698
N	-2.289160	1.467677	1.345207
N	-0.230377	1.520622	0.636553
O	-3.944061	-0.148516	-2.022847
O	3.951436	0.142770	-2.010212
P	-1.049868	-2.813886	-1.871968
P	1.053465	2.807148	-1.878114
C	0.753135	-2.993241	-2.224872
H	0.941106	-3.804294	-2.930617
H	1.127315	-2.050370	-2.622348
H	1.273536	-3.190104	-1.288349
C	-1.804664	-2.804748	-3.555392
H	-1.497869	-3.679197	-4.131916
H	-2.890729	-2.790329	-3.463263
H	-1.494525	-1.897768	-4.073868
C	-1.497895	-4.500324	-1.254305
H	-2.573821	-4.547441	-1.086995
H	-1.203777	-5.274569	-1.966205
H	-0.991219	-4.675113	-0.305547
C	-0.748691	2.983551	-2.236706
H	-0.935383	3.791707	-2.946100
H	-1.121202	2.038860	-2.631427
H	-1.271684	3.183732	-1.302354
C	1.497843	4.496076	-1.264572
H	2.573128	4.544653	-1.093608
H	1.205607	5.267736	-1.980057
H	0.987652	4.673539	-0.318197
C	1.813246	2.793163	-3.559224
H	1.506892	3.664953	-4.139989
H	2.899048	2.780754	-3.463728
H	1.506122	1.883715	-4.075167

5_{H-H}

Zero-point correction= 0.696722 (Hartree/Particle)
Thermal correction to Energy= 0.747636
Thermal correction to Enthalpy= 0.748580
Thermal correction to Gibbs Free Energy= 0.607889
Sum of electronic and zero-point Energies= -2507.128830
Sum of electronic and thermal Energies= -2507.077916
Sum of electronic and thermal Enthalpies= -2507.076972
Sum of electronic and thermal Free Energies= -2507.217663

C	-1.411295	0.616942	-0.722758
C	-0.555051	1.604660	-2.524366
H	0.184663	1.820755	-3.274773
C	-1.800240	2.121611	-2.360974
H	-2.349478	2.862797	-2.912921
C	-3.660021	1.734399	-0.753874
C	-4.752494	1.366020	-1.551460
C	-6.038992	1.597638	-1.064439
H	-6.887734	1.303392	-1.672492
C	-6.260080	2.179952	0.180024
C	-5.151616	2.554488	0.936106
H	-5.303399	3.027864	1.900335
C	-3.846715	2.351947	0.491383
C	-4.574068	0.724397	-2.904142
H	-5.460268	0.150303	-3.176615
H	-3.710757	0.061518	-2.929376
H	-4.414491	1.475476	-3.681879
C	-7.657017	2.377727	0.710496
H	-8.393267	2.388423	-0.094590
H	-7.743540	3.315732	1.262402
H	-7.929610	1.570263	1.396603
C	-2.687297	2.776114	1.347681
H	-2.990412	3.549530	2.054387
H	-1.854064	3.147776	0.751479
H	-2.310816	1.915067	1.905490
C	-1.135370	-1.510801	2.651266
C	1.391972	0.640685	0.715446
C	1.771148	2.171028	2.332092
H	2.315993	2.922409	2.874537
C	0.528157	1.650504	2.500883
H	-0.214724	1.875637	3.245533
C	3.640644	1.753533	0.740298
C	4.729084	1.404367	1.550896
C	6.018638	1.614110	1.058558
H	6.864375	1.331975	1.676363
C	6.245055	2.155921	-0.201931
C	5.139303	2.513388	-0.971856
H	5.295980	2.952918	-1.951358
C	3.833657	2.334090	-0.523004
C	4.551716	0.811608	2.925984
H	5.406354	0.186484	3.187641
H	3.647010	0.211867	3.001599
H	4.472972	1.595451	3.684192
C	7.642010	2.360167	-0.729976
H	8.393668	2.023218	-0.015155
H	7.831237	3.415448	-0.943391
H	7.793787	1.809599	-1.661920
C	2.677625	2.743876	-1.391428
H	2.291812	1.872084	-1.925302
H	2.988130	3.494659	-2.119044
H	1.848942	3.140594	-0.804982
C	1.173622	-1.512378	-2.645430
Ir	-1.301115	-0.589264	1.028247
Ir	1.295862	-0.592494	-1.017566
N	-0.332813	0.692170	-1.516726
N	-2.336053	1.496680	-1.241061
N	2.312918	1.530643	1.224008
N	0.312058	0.721746	1.506997
O	1.097867	-2.056380	-3.663906
O	-1.032059	-2.060281	3.664364
P	2.819818	-2.066019	-0.178899
P	-2.792428	-2.099285	0.196948
C	2.723581	-3.772401	-0.881399
H	3.436976	-4.437470	-0.391755
H	1.712740	-4.154134	-0.740509
H	2.931706	-3.739524	-1.950561
C	2.593655	-2.396867	1.620824
H	3.307118	-3.138881	1.982997
H	2.709578	-1.469800	2.176423
H	1.573916	-2.747349	1.775193
C	4.623961	-1.707577	-0.337606
H	4.851544	-0.748908	0.121519
H	5.218956	-2.493096	0.133359
H	4.873995	-1.645693	-1.396569
C	-2.700471	-3.786412	0.944560
H	-3.392605	-4.471354	0.451857
H	-1.682068	-4.160353	0.842425
H	-2.939431	-3.728934	2.006218
C	-4.603046	-1.752776	0.300161
H	-4.826377	-0.802905	-0.179101
H	-5.179700	-2.550278	-0.173507
H	-4.881399	-1.676125	1.351135
C	-2.515366	-2.473773	-1.587252
H	-3.226365	-3.215381	-1.954992
H	-2.598689	-1.558187	-2.167941
H	-1.496047	-2.840437	-1.700853

6

Zero-point correction= 0.738172 (Hartree/Particle)
Thermal correction to Energy= 0.792038
Thermal correction to Enthalpy= 0.792983
Thermal correction to Gibbs Free Energy= 0.650252
Sum of electronic and zero-point Energies= -2558.512972
Sum of electronic and thermal Energies= -2558.459106
Sum of electronic and thermal Enthalpies= -2558.458162
Sum of electronic and thermal Free Energies= -2558.600892

C	-1.531049	0.689260	-0.766413
C	-0.304699	1.754685	-2.278514
H	0.582515	1.971017	-2.848345
C	-1.534173	2.338004	-2.284449
H	-1.941204	3.157376	-2.849943
C	-3.622539	2.040365	-0.945116
C	-4.720631	1.423924	-1.544754
C	-5.994452	1.808698	-1.123894
H	-6.859883	1.340011	-1.579548
C	-6.180486	2.775614	-0.139292
C	-5.055006	3.374853	0.428193
H	-5.185317	4.131218	1.194604
C	-3.766201	3.021852	0.042354
C	-4.542490	0.354573	-2.590385
H	-5.452086	0.232693	-3.178876
H	-4.316976	-0.608309	-2.126691
H	-3.720519	0.586901	-3.268007
C	-7.563467	3.152004	0.327649
H	-8.322219	2.878527	-0.406874
H	-7.641861	4.224898	0.514090
H	-7.808749	2.639808	1.262664
C	-2.554349	3.646672	0.679762
H	-2.843232	4.368526	1.443383
H	-1.933865	4.158388	-0.059248
H	-1.922555	2.886876	1.142782
C	-2.651316	-1.929796	-0.234931
C	0.929571	0.511603	1.164609
C	1.014977	1.892832	2.931281
H	1.466905	2.582452	3.621814
C	-0.250160	1.403493	2.828243
H	-1.114038	1.589084	3.441943
C	3.109352	1.676071	1.607601
C	4.140579	0.884255	2.110764
C	5.450896	1.256826	1.813019
H	6.265679	0.646203	2.186948
C	5.736420	2.381664	1.045074
C	4.676054	3.156611	0.578112
H	4.882956	4.027237	-0.034109
C	3.353426	2.818334	0.838804
C	3.864844	-0.355455	2.919748
H	4.716887	-0.599223	3.555325
H	3.688043	-1.211469	2.265367
H	2.985814	-0.239520	3.554188
C	7.156528	2.729756	0.681081
H	7.868275	2.308236	1.392847
H	7.307255	3.810530	0.646865
H	7.402739	2.332621	-0.308010
C	2.214244	3.601244	0.245917
H	1.684338	2.981530	-0.481345
H	2.579581	4.490031	-0.267878
H	1.490469	3.911002	1.002781
C	1.880749	-2.090868	0.315280
Ir	-1.599670	-0.760526	0.736462
Ir	0.963723	-0.728918	-0.524651
N	-0.332672	0.745840	-1.343763
N	-2.298468	1.662650	-1.334584
N	1.745021	1.330355	1.889892
N	-0.275846	0.557867	1.742993
O	2.437047	-2.966652	0.828821
O	-3.321515	-2.659840	-0.830714
P	0.447477	-2.149076	-2.367472
P	-1.141253	-2.383483	2.426068
C	-0.927531	-1.540510	-3.428430
H	-1.152659	-2.264915	-4.212205
H	-1.812104	-1.370267	-2.820047
H	-0.631654	-0.592645	-3.875234
C	-0.088429	-3.852378	-1.903290
H	-0.312421	-4.445047	-2.791754
H	0.715149	-4.333948	-1.345387
H	-0.970088	-3.799712	-1.270716
C	1.777938	-2.504331	-3.588050
H	2.634700	-2.928381	-3.064998
H	1.422087	-3.200408	-4.350175
H	2.105234	-1.570952	-4.041619
C	0.306374	-2.010325	3.497525
H	0.448637	-2.809411	4.226219
H	1.199130	-1.911933	2.887299
H	0.137117	-1.065936	4.012254
C	-2.471700	-2.686981	3.670386
H	-3.376585	-3.021534	3.162987
H	-2.158053	-3.445597	4.389241
H	-2.696405	-1.760028	4.196595
C	-0.799511	-4.089132	1.818872
H	-0.656182	-4.774780	2.655593
H	-1.639558	-4.429909	1.213364
H	0.096617	-4.078958	1.203756
I	3.244644	0.454363	-1.935473
C	-3.334721	0.050671	1.764994
H	-3.024912	0.939149	2.317236
H	-4.105951	0.348421	1.058861
H	-3.777328	-0.655164	2.466516

6isomer1

Zero-point correction= 0.735811 (Hartree/Particle)
Thermal correction to Energy= 0.790634
Thermal correction to Enthalpy= 0.791578
Thermal correction to Gibbs Free Energy= 0.645139
Sum of electronic and zero-point Energies= -2558.484535
Sum of electronic and thermal Energies= -2558.429712
Sum of electronic and thermal Enthalpies= -2558.428768
Sum of electronic and thermal Free Energies= -2558.575207

C	-1.647931	-0.743857	0.781265
C	-0.720164	-2.015718	2.352362
H	0.067343	-2.387898	2.984629
C	-2.028372	-2.381942	2.271760
H	-2.598911	-3.127341	2.796399
C	-3.947791	-1.689310	0.815461
C	-4.972177	-1.025550	1.494967
C	-6.268037	-1.143662	0.995577
H	-7.075836	-0.633059	1.508495
C	-6.549424	-1.895236	-0.144150
C	-5.500458	-2.552125	-0.785144
H	-5.704853	-3.141232	-1.672288
C	-4.190238	-2.463985	-0.322889
C	-4.681952	-0.204178	2.723129
H	-5.600251	0.208606	3.140318
H	-4.013240	0.624397	2.486068
H	-4.192504	-0.799939	3.496488
C	-7.950856	-1.967906	-0.693880
H	-8.695965	-1.810640	0.087636
H	-8.146253	-2.934696	-1.161237
H	-8.105088	-1.198627	-1.456306
C	-3.059288	-3.164006	-1.028261
H	-3.425384	-3.730690	-1.883880
H	-2.539551	-3.851626	-0.356691
H	-2.312467	-2.450352	-1.379434
C	-3.020387	0.383251	-1.688038
C	0.998278	-0.812865	-0.971770
C	1.143657	-2.093081	-2.811184
H	1.589931	-2.791406	-3.496764
C	-0.053902	-1.445269	-2.816750
H	-0.853126	-1.479494	-3.537715
C	3.055998	-2.216779	-1.210670
C	4.243303	-1.683019	-1.720081
C	5.445899	-2.207737	-1.250080
H	6.376698	-1.797401	-1.626029
C	5.483018	-3.237703	-0.311936
C	4.276870	-3.767208	0.143062
H	4.289013	-4.579234	0.861759
C	3.050992	-3.275441	-0.297134
C	4.221761	-0.586042	-2.748971
H	5.234605	-0.298024	-3.029955
H	3.712859	0.296830	-2.361136
H	3.691562	-0.900025	-3.651102
C	6.797401	-3.746515	0.221792
H	7.596187	-3.639645	-0.514113
H	6.733496	-4.798629	0.505145
H	7.093647	-3.184056	1.112211
C	1.753014	-3.861853	0.190416
H	1.141029	-3.112321	0.691940
H	1.932764	-4.682336	0.884812
H	1.155605	-4.240281	-0.642756
C	2.468276	-0.726036	1.734152
Ir	-1.444484	0.706536	-0.671682
Ir	1.030421	0.226061	0.831379
N	-0.520129	-1.013175	1.428584
N	-2.602389	-1.578568	1.289673
N	1.799406	-1.692098	-1.652533
N	-0.117025	-0.670209	-1.681248
O	3.233679	-1.316173	2.351669
O	-3.951850	0.181986	-2.333625
P	0.619431	1.686732	2.666506
P	-0.708572	2.486249	-2.070349
C	-0.724438	1.138182	3.806655
H	-0.912459	1.890735	4.573737
H	-1.636367	0.947249	3.246272
H	-0.420897	0.205353	4.280764
C	0.227831	3.446425	2.302301
H	0.024915	3.989150	3.227229
H	1.090199	3.882282	1.800403
H	-0.629627	3.500454	1.640237
C	2.047757	1.870467	3.821275
H	2.904504	2.227650	3.249909
H	1.819885	2.574123	4.623804
H	2.295870	0.898971	4.248506
C	0.634259	2.061225	-3.252521
H	0.877094	2.919522	-3.880848
H	1.514286	1.755097	-2.692576
H	0.305908	1.228239	-3.872971
C	-2.035884	3.103988	-3.203073
H	-2.883729	3.439934	-2.605098
H	-1.676226	3.930285	-3.818586
H	-2.372850	2.291846	-3.847051
C	-0.163068	4.066326	-1.301102
H	0.124376	4.778246	-2.076798
H	-0.985603	4.482916	-0.721334
H	0.683651	3.878239	-0.647760
I	2.930162	2.086730	-0.113173
C	-2.563258	2.071483	0.568679
H	-3.627240	1.827642	0.552833
H	-2.243617	2.019603	1.606745
H	-2.479815	3.116135	0.258227

6isomer2

Zero-point correction= 0.737087 (Hartree/Particle)
Thermal correction to Energy= 0.791396
Thermal correction to Enthalpy= 0.792340
Thermal correction to Gibbs Free Energy= 0.648486
Sum of electronic and zero-point Energies= -2558.477573
Sum of electronic and thermal Energies= -2558.423265
Sum of electronic and thermal Enthalpies= -2558.422320
Sum of electronic and thermal Free Energies= -2558.566174

C	1.511351	0.576165	0.904501
C	0.423944	1.818793	2.407191
H	-0.404025	2.144961	3.007159
C	1.688169	2.309621	2.337687
H	2.172162	3.125725	2.844003
C	3.688667	1.829226	0.971655
C	4.770853	1.436980	1.764159
C	6.049110	1.821366	1.361271
H	6.900224	1.523672	1.963628
C	6.261502	2.555979	0.195591
C	5.155145	2.923597	-0.570984
H	5.303892	3.503734	-1.475299
C	3.856814	2.584561	-0.193614
C	4.558330	0.592892	2.991672
H	5.506830	0.362470	3.476110
H	4.064970	-0.345850	2.730947
H	3.914427	1.093079	3.717730
C	7.657479	2.910979	-0.247978
H	8.335490	2.998828	0.602282
H	7.674657	3.854352	-0.796197
H	8.060945	2.139480	-0.910782
C	2.661341	3.040136	-0.987660
H	2.968654	3.567381	-1.890786
H	2.039954	3.715399	-0.393741
H	2.021226	2.204439	-1.270909
C	0.855921	-2.733946	-1.195787
C	-0.971787	0.463239	-0.990955
C	-0.947183	1.592015	-2.940798
H	-1.320059	2.249766	-3.705937
C	0.205630	0.881234	-2.828790
H	1.031277	0.816387	-3.513814
C	-2.906682	2.003226	-1.504800
C	-4.083917	1.584327	-2.134155
C	-5.242655	2.326879	-1.910044
H	-6.164235	2.012420	-2.387356
C	-5.253117	3.443878	-1.075534
C	-4.058279	3.832422	-0.470659
H	-4.045483	4.708543	0.168637
C	-2.869060	3.137308	-0.686585
C	-4.100677	0.356068	-3.001666
H	-5.101376	0.169399	-3.391030
H	-3.778018	-0.520489	-2.435056
H	-3.415602	0.453664	-3.846251
C	-6.534838	4.189327	-0.805424
H	-7.175775	4.212790	-1.688559
H	-6.342104	5.218140	-0.497316
H	-7.101780	3.705924	-0.004056
C	-1.567620	3.614006	-0.098983
H	-1.069414	2.830758	0.469832
H	-1.722549	4.474774	0.551875
H	-0.874210	3.906866	-0.891846
C	-0.708922	-1.826977	2.247651
Ir	1.376806	-1.100091	-0.375914
Ir	-1.082822	-0.591711	0.842748
N	0.339966	0.772034	1.518867
N	2.360801	1.528571	1.402216
N	-1.680469	1.326539	-1.789538
N	0.174381	0.207388	-1.631221
O	-0.455284	-2.591779	3.062391
O	0.616810	-3.761039	-1.644593
P	-3.138193	0.198470	1.910945
P	3.460450	-1.132573	-1.545583
C	-3.129183	1.788582	2.863809
H	-4.117960	1.980028	3.285606
H	-2.409584	1.723198	3.679536
H	-2.857434	2.613175	2.207478
C	-3.628026	-0.996829	3.234598
H	-4.550923	-0.681560	3.725023
H	-3.772808	-1.973651	2.772752
H	-2.832344	-1.077903	3.974727
C	-4.728222	0.350126	0.992090
H	-4.831823	-0.494622	0.314253
H	-5.559806	0.359968	1.699633
H	-4.735669	1.271317	0.420507
C	3.711564	0.021016	-2.970376
H	4.709254	-0.109825	-3.394205
H	2.970385	-0.197377	-3.738233
H	3.593457	1.051272	-2.642727
C	5.056105	-0.937063	-0.638207
H	4.992043	-1.421843	0.333452
H	5.872109	-1.379111	-1.212571
H	5.259681	0.117543	-0.488796
C	3.742525	-2.758886	-2.382484
H	4.700754	-2.765070	-2.904926
H	3.730602	-3.553455	-1.636471
H	2.940249	-2.951034	-3.093751
I	-2.623717	-2.558897	-0.428618
C	2.239471	-2.276677	1.218161
H	3.270984	-2.591805	1.034525
H	2.235382	-1.701676	2.145501
H	1.662240	-3.182938	1.403616

6isomer3

Zero-point correction= 0.737440 (Hartree/Particle)
Thermal correction to Energy= 0.791716
Thermal correction to Enthalpy= 0.792660
Thermal correction to Gibbs Free Energy= 0.648034
Sum of electronic and zero-point Energies= -2558.481332
Sum of electronic and thermal Energies= -2558.427056
Sum of electronic and thermal Enthalpies= -2558.426112
Sum of electronic and thermal Free Energies= -2558.570739

C	-1.316320	0.116618	-0.935921
C	-0.097805	0.315175	-2.790893
H	0.770084	0.231652	-3.417538
C	-1.340761	0.767596	-3.095875
H	-1.764051	1.160976	-4.002921
C	-3.429647	1.141893	-1.841086
C	-4.490221	0.365544	-2.317304
C	-5.767980	0.922580	-2.297365
H	-6.602242	0.332827	-2.660901
C	-6.001914	2.206825	-1.806922
C	-4.917466	2.949502	-1.339336
H	-5.083117	3.953724	-0.964287
C	-3.619082	2.441597	-1.359918
C	-4.253055	-1.036346	-2.809172
H	-5.186991	-1.505877	-3.117020
H	-3.797437	-1.647667	-2.027931
H	-3.563996	-1.052459	-3.655873
C	-7.401392	2.763204	-1.745365
H	-8.017889	2.382683	-2.561380
H	-7.401092	3.853055	-1.798483
H	-7.889218	2.479860	-0.807765
C	-2.442681	3.271998	-0.918211
H	-2.771663	4.198428	-0.446961
H	-1.814559	3.532898	-1.774313
H	-1.803480	2.729259	-0.222189
C	-0.818620	-1.276224	2.739763
C	1.101152	1.058887	0.827089
C	1.146046	3.102853	1.764898
H	1.570012	4.051385	2.043441
C	-0.080219	2.554197	1.962138
H	-0.934091	2.962360	2.470955
C	3.195360	2.478303	0.575340
C	4.311059	2.183096	1.362803
C	5.555305	2.636608	0.924224
H	6.432596	2.418839	1.523661
C	5.697535	3.373232	-0.251334
C	4.558996	3.645799	-1.008473
H	4.655594	4.209635	-1.929989
C	3.298359	3.201483	-0.617314
C	4.172929	1.398911	2.639955
H	5.098280	1.424747	3.214980
H	3.932181	0.354204	2.435834
H	3.366614	1.789614	3.262766
C	7.058243	3.825168	-0.715431
H	7.720830	4.027127	0.127756
H	6.993043	4.729644	-1.322279
H	7.533357	3.052555	-1.327478
C	2.075034	3.456602	-1.457939
H	1.572448	2.518756	-1.707532
H	2.336111	3.969552	-2.383491
H	1.342737	4.065143	-0.922291
C	2.071675	-1.525003	1.490577
Ir	-1.327214	-0.461658	1.105306
Ir	1.224200	-0.827781	-0.018321
N	-0.105617	-0.073432	-1.473227
N	-2.097266	0.637991	-1.934334
N	1.886407	2.161521	1.054833
N	-0.086660	1.309144	1.382060
O	2.603696	-1.943326	2.420258
O	-0.499326	-1.815529	3.702473
P	3.234571	-1.001834	-1.457371
P	-3.423751	0.252404	1.961560
C	2.952936	-1.526973	-3.211862
H	3.873057	-1.952402	-3.617628
H	2.154949	-2.263167	-3.260149
H	2.686063	-0.654688	-3.808774
C	4.350750	-2.323131	-0.809745
H	5.205017	-2.463598	-1.474688
H	4.707093	-2.032632	0.179212
H	3.789211	-3.251216	-0.721568
C	4.462707	0.342029	-1.761749
H	4.884469	0.696472	-0.825608
H	5.265730	-0.039878	-2.396288
H	3.982782	1.179465	-2.261758
C	-3.602677	2.019936	2.474789
H	-4.613953	2.213015	2.837777
H	-2.892867	2.232475	3.273650
H	-3.393380	2.671728	1.629227
C	-4.991772	-0.018078	1.028273
H	-4.971968	-0.990849	0.542289
H	-5.842903	0.031308	1.710072
H	-5.099108	0.749910	0.270753
C	-3.814378	-0.617094	3.545810
H	-4.776031	-0.290804	3.946009
H	-3.845379	-1.690562	3.359990
H	-3.030571	-0.419653	4.276145
I	0.706199	-3.492139	-0.932543
C	-2.298374	-2.308265	0.572106
H	-3.310934	-2.405318	0.976978
H	-2.355460	-2.395969	-0.511249
H	-1.724002	-3.161811	0.924441

6isomer4

Zero-point correction= 0.736805 (Hartree/Particle)
Thermal correction to Energy= 0.791184
Thermal correction to Enthalpy= 0.792128
Thermal correction to Gibbs Free Energy= 0.647601
Sum of electronic and zero-point Energies= -2558.486471
Sum of electronic and thermal Energies= -2558.432092
Sum of electronic and thermal Enthalpies= -2558.431148
Sum of electronic and thermal Free Energies= -2558.575675

C	-1.330230	-0.047251	-0.964977
C	-0.263721	-0.835519	-2.755062
H	0.562026	-1.209080	-3.330455
C	-1.560126	-0.672259	-3.121147
H	-2.076511	-0.857245	-4.046080
C	-3.628522	0.049942	-1.952029
C	-4.469888	-0.903085	-1.370025
C	-5.840540	-0.629996	-1.340745
H	-6.502701	-1.356550	-0.881573
C	-6.376317	0.531196	-1.887928
C	-5.508228	1.438177	-2.498938
H	-5.910051	2.340811	-2.946968
C	-4.135377	1.214351	-2.547114
C	-3.959578	-2.210491	-0.823366
H	-4.340275	-2.386565	0.183160
H	-2.874923	-2.260482	-0.779673
H	-4.306524	-3.034382	-1.452822
C	-7.853086	0.820358	-1.801060
H	-8.434918	-0.098019	-1.710903
H	-8.206152	1.362173	-2.680359
H	-8.076346	1.437682	-0.925553
C	-3.214030	2.194010	-3.227746
H	-3.709701	3.152919	-3.381000
H	-2.898256	1.818391	-4.204183
H	-2.306776	2.357095	-2.645051
C	-2.161100	-1.050876	1.774009
C	1.344192	1.289114	0.244826
C	1.703677	3.504308	0.371557
H	2.270871	4.416454	0.320090
C	0.401133	3.253516	0.663473
H	-0.376302	3.942464	0.941558
C	3.665718	2.161369	-0.270680
C	4.642357	1.916522	0.696273
C	5.978974	1.920222	0.286978
H	6.749299	1.730182	1.026770
C	6.346849	2.180012	-1.029606
C	5.340065	2.431314	-1.963905
H	5.609611	2.627861	-2.995979
C	3.996212	2.417222	-1.608768
C	4.296198	1.652014	2.137667
H	4.998419	2.161233	2.799759
H	4.351853	0.584956	2.360936
H	3.288210	1.979234	2.384691
C	7.793012	2.150430	-1.452155
H	8.460637	2.323149	-0.606980
H	8.001774	2.906278	-2.211467
H	8.049412	1.177169	-1.881289
C	2.912841	2.632451	-2.632537
H	2.208233	1.797136	-2.632371
H	3.335391	2.732188	-3.632189
H	2.328730	3.529569	-2.416253
C	1.973800	-0.992578	1.862834
Ir	-1.206765	0.407288	1.122335
Ir	1.157414	-0.761199	0.205187
N	-0.143773	-0.450894	-1.439677
N	-2.220995	-0.183012	-1.997609
N	2.292192	2.269544	0.111364
N	0.203246	1.896915	0.571973
O	2.479533	-1.152661	2.882807
O	-2.788073	-1.878211	2.267996
P	3.040684	-1.783820	-1.041951
P	-2.981409	1.945622	1.490416
C	2.607640	-2.895924	-2.457282
H	3.444791	-3.564820	-2.666466
H	1.722364	-3.479180	-2.212115
H	2.416805	-2.294034	-3.345581
C	3.965268	-2.928556	0.074084
H	4.751764	-3.451021	-0.473591
H	4.410173	-2.356464	0.888658
H	3.263531	-3.646954	0.495193
C	4.433038	-0.840034	-1.800760
H	4.944352	-0.251861	-1.043105
H	5.141841	-1.531935	-2.261352
H	4.048861	-0.162672	-2.559936
C	-3.281586	3.208871	0.177705
H	-3.895552	4.026124	0.561029
H	-2.334681	3.601500	-0.187437
H	-3.799465	2.732000	-0.648807
C	-4.662283	1.233019	1.738422
H	-4.630264	0.525956	2.567435
H	-5.390627	2.016471	1.956188
H	-4.959986	0.698768	0.837718
C	-2.795282	3.014093	2.988561
H	-3.605679	3.743421	3.038999
H	-2.804752	2.395402	3.883619
H	-1.836332	3.530155	2.952495
I	0.104862	-3.419755	0.376130
C	-0.528150	0.718390	3.175288
H	-1.329481	0.708026	3.918156
H	0.177898	-0.053794	3.472744
H	-0.021336	1.684585	3.245400

$3_{H\text{-}H}$+CO

Zero-point correction= 0.833748 (Hartree/Particle)
Thermal correction to Energy= 0.883861
Thermal correction to Enthalpy= 0.884805
Thermal correction to Gibbs Free Energy= 0.750837
Sum of electronic and zero-point Energies= -2208.876991
Sum of electronic and thermal Energies= -2208.826878
Sum of electronic and thermal Enthalpies= -2208.825934
Sum of electronic and thermal Free Energies= -2208.959902

C	-0.050634	-1.144191	-0.676335
C	1.316484	-1.881611	-2.287527
H	2.221293	-1.938537	-2.864008
C	0.154592	-2.549339	-2.448647
H	-0.157643	-3.292025	-3.160724
C	-1.960554	-2.727426	-1.220018
C	-1.993240	-3.884881	-0.427809
C	-3.222128	-4.499397	-0.211066
H	-3.262262	-5.384849	0.414348
C	-4.398286	-4.011064	-0.781855
C	-4.314907	-2.903235	-1.617430
H	-5.213546	-2.523604	-2.091615
C	-3.104775	-2.254009	-1.864064
C	-0.727884	-4.490101	0.123863
H	-0.211513	-5.063415	-0.651176
H	-0.947862	-5.165221	0.950725
H	-0.030481	-3.732474	0.475985
C	-5.725246	-4.660338	-0.483692
H	-5.621118	-5.738582	-0.348340
H	-6.444672	-4.485523	-1.285269
H	-6.154689	-4.254973	0.437362
C	-3.058110	-1.103666	-2.832522
H	-3.081651	-1.473875	-3.861645
H	-2.154633	-0.515275	-2.713654
H	-3.913955	-0.443968	-2.690688
C	0.064798	-1.404283	2.252196
H	0.690144	-2.121015	1.732580
C	-1.346443	-1.703014	2.207044
H	-1.618718	-2.605014	1.679358
C	-2.297643	-1.389993	3.365812
H	-3.104168	-2.126424	3.366724
H	-1.777071	-1.524439	4.318322
C	-2.923396	0.015690	3.285490
H	-3.194548	0.384093	4.284098
H	-3.854141	-0.044426	2.718894
C	-2.045922	1.039055	2.595596
H	-2.577992	1.867037	2.145213
C	-0.689275	1.205382	2.836226
H	-0.238946	2.131625	2.508230
C	0.064513	0.478822	3.938919
H	0.786253	1.172825	4.376453
H	-0.632244	0.223900	4.738603
C	0.784457	-0.769637	3.422721
H	0.943198	-1.493598	4.235675
H	1.770206	-0.476527	3.068603
C	0.175914	1.634810	0.078862
C	1.800826	3.060299	-0.439866
H	2.807828	3.387265	-0.633555
C	0.651098	3.765263	-0.545455
H	0.449667	4.782631	-0.831288
C	-1.733688	3.143914	-0.555029
C	-2.564699	3.852399	0.312484
C	-3.897433	4.040811	-0.062320
H	-4.559690	4.569816	0.614976
C	-4.393797	3.571862	-1.273569
C	-3.514596	2.928793	-2.146743
H	-3.878698	2.581149	-3.107541
C	-2.184122	2.711316	-1.812622
C	-2.051228	4.456556	1.593912
H	-2.812489	4.428906	2.374960
H	-1.781147	5.504935	1.436367
H	-1.163180	3.946253	1.957767
C	-5.849855	3.724476	-1.629116
H	-5.981884	3.918882	-2.695391
H	-6.315504	4.540423	-1.074179
H	-6.398652	2.808018	-1.392793
C	-1.234057	2.039788	-2.767330
H	-1.736798	1.770465	-3.695457
H	-0.803676	1.140271	-2.325103
H	-0.395441	2.697753	-3.007456
C	4.315769	1.200520	0.726095
H	3.998632	2.235753	0.714624
C	5.063658	0.760323	-0.367119
H	5.250218	1.494181	-1.142806
C	6.047212	-0.400761	-0.332808
H	6.864470	-0.183154	-1.022896
H	6.494475	-0.451037	0.661104
C	5.393097	-1.742854	-0.704284
H	5.928819	-2.574647	-0.228483
H	5.485853	-1.892322	-1.783004
C	3.914717	-1.783547	-0.366820
H	3.400067	-2.593562	-0.876714
C	3.356411	-1.416652	0.918499
H	2.486366	-1.985839	1.215755
C	4.228351	-0.947621	2.083054
H	5.219898	-1.401894	2.025135
H	3.803070	-1.295771	3.025644
C	4.326704	0.583295	2.108383

H	3.460687	0.985460	2.637886
H	5.215237	0.928901	2.654148
N	1.194580	-1.035106	-1.196772
N	-0.689546	-2.099815	-1.448550
N	1.502168	1.761513	-0.061905
N	-0.366721	2.879956	-0.220897
Ir	-0.907258	-0.034548	0.866434
Ir	2.905580	0.070576	-0.585577
C	3.097783	0.779900	-2.399061
C	-2.674368	0.209785	0.078552
O	-3.812713	0.272298	-0.086238
O	3.386199	1.071308	-3.471575

$4_{H-H}+CO$

Zero-point correction= 0.498203 (Hartree/Particle)
Thermal correction to Energy= 0.544391
Thermal correction to Enthalpy= 0.545335
Thermal correction to Gibbs Free Energy= 0.411788
Sum of electronic and zero-point Energies= -2038.427280
Sum of electronic and thermal Energies= -2038.381093
Sum of electronic and thermal Enthalpies= -2038.380148
Sum of electronic and thermal Free Energies= -2038.513696

C	0.295194	1.405734	0.382945
C	1.770400	2.340347	1.771923
H	2.721146	2.468867	2.257898
C	0.603249	3.003693	1.949766
H	0.328205	3.817920	2.596025
C	-1.655045	2.872977	0.906445
C	-1.897120	3.946983	0.045524
C	-3.217841	4.357121	-0.130846
H	-3.424140	5.179076	-0.807470
C	-4.274299	3.732033	0.527822
C	-3.987536	2.681027	1.398720
H	-4.798712	2.187853	1.922663
C	-2.684827	2.237962	1.607106
C	-0.767784	4.645913	-0.664961
H	-0.234707	5.315452	0.015266
H	-1.142770	5.238713	-1.498536
H	-0.034133	3.937897	-1.051049
C	-5.699137	4.155120	0.278422
H	-5.758394	5.200989	-0.026595
H	-6.315660	4.023968	1.169400
H	-6.144685	3.553529	-0.519377
C	-2.383124	1.105364	2.547952
H	-1.722053	1.427402	3.356112
H	-1.873242	0.298623	2.023661
H	-3.297118	0.706109	2.986104
C	-1.294074	1.526230	-2.152603
C	0.554617	-0.531353	-2.506476
C	0.341121	-1.455450	0.047461
C	1.959615	-2.640768	1.015519
H	2.952998	-2.850482	1.369182
C	0.831799	-3.381497	1.111870
H	0.635847	-4.344433	1.547698
C	-1.534242	-3.078223	0.449722
C	-2.026683	-3.640612	-0.733781
C	-3.359448	-4.042182	-0.755155
H	-3.761220	-4.469133	-1.667540
C	-4.188360	-3.905464	0.357297
C	-3.648140	-3.378944	1.527970
H	-4.276858	-3.278722	2.405938
C	-2.320475	-2.965268	1.601245
C	-1.158273	-3.782569	-1.953235
H	-1.057497	-2.820160	-2.459136
H	-1.590596	-4.495219	-2.655512
H	-0.152007	-4.116349	-1.695954
C	-5.643222	-4.288730	0.281841
H	-6.040403	-4.550591	1.263736
H	-5.798374	-5.137246	-0.386775
H	-6.237466	-3.454820	-0.103100
C	-1.757466	-2.430632	2.892698
H	-2.545377	-1.993274	3.505734
H	-0.992955	-1.673967	2.723434
H	-1.291210	-3.231975	3.472955
C	4.263924	-1.121695	-0.740501
C	4.201960	1.535135	-0.265414
N	1.571150	1.367623	0.806825
N	-0.307342	2.418150	1.081272
N	1.650062	-1.461055	0.362946
N	-0.171149	-2.641075	0.504439
Ir	-0.602287	0.077541	-1.070462
Ir	3.028941	0.092744	-0.012649
O	4.906445	2.427313	-0.407362
O	5.005865	-1.876248	-1.179323
O	1.233409	-0.929738	-3.343135
O	-1.681726	2.364836	-2.823952
C	-2.417104	-0.403392	-0.535099
C	3.942065	-0.097668	3.290037
O	3.908178	-1.214428	3.450437
O	-3.534482	-0.552311	-0.316522

$4_{H\text{-}T}$+CO

Zero-point correction= 0.498835 (Hartree/Particle)
Thermal correction to Energy= 0.544121
Thermal correction to Enthalpy= 0.545066
Thermal correction to Gibbs Free Energy= 0.414924
Sum of electronic and zero-point Energies= -2038.424281
Sum of electronic and thermal Energies= -2038.378994
Sum of electronic and thermal Enthalpies= -2038.378050
Sum of electronic and thermal Free Energies= -2038.508191

C	-1.312785	-0.064440	0.915916
C	-0.187141	-0.846926	2.649159
H	0.643292	-0.944211	3.326659
C	-1.380392	-1.494203	2.647311
H	-1.799748	-2.245754	3.292393
C	-3.335402	-1.527357	1.118003
C	-4.518333	-0.878374	1.478224
C	-5.719256	-1.424968	1.031882
H	-6.648737	-0.931537	1.293583
C	-5.755033	-2.580151	0.252452
C	-4.551049	-3.199579	-0.081358
H	-4.565376	-4.091547	-0.697849
C	-3.326116	-2.686924	0.334636
C	-4.481740	0.392328	2.282431
H	-5.484337	0.692660	2.585633
H	-4.044807	1.200771	1.691530
H	-3.866391	0.283179	3.177370
C	-7.064250	-3.120129	-0.261754
H	-7.888114	-2.880963	0.412504
H	-7.028783	-4.203623	-0.386641
H	-7.299711	-2.683941	-1.236969
C	-2.024709	-3.314003	-0.092013
H	-2.200925	-4.194723	-0.708901
H	-1.418019	-3.611137	0.765799
H	-1.425269	-2.604812	-0.669214
C	-1.735486	2.552215	0.397520
C	-1.769881	2.215659	-2.383158
C	1.312786	-0.064479	-0.915891
C	1.380427	-1.494209	-2.647312
H	1.799790	-2.245762	-3.292387
C	0.187184	-0.846915	-2.649185
H	-0.643250	-0.944218	-3.326682
C	3.335409	-1.527378	-1.117963
C	4.518327	-0.878543	-1.478425
C	5.719281	-1.425115	-1.032079
H	6.648751	-0.931779	-1.293987
C	5.755061	-2.580099	-0.252392
C	4.551054	-3.199397	0.081672
H	4.565411	-4.091230	0.698362
C	3.326131	-2.686796	-0.334340
C	4.481705	0.392024	-2.282847
H	5.484301	0.692371	-2.586037
H	4.044695	1.200539	-1.692100
H	3.866419	0.282704	-3.177809
C	7.064223	-3.120135	0.261889
H	7.888596	-2.878595	-0.410884
H	7.029586	-4.203971	0.384100
H	7.298216	-2.686150	1.238440
C	2.024714	-3.313747	0.092460
H	1.425470	-2.604557	0.669866
H	2.200910	-4.194584	0.709189
H	1.417840	-3.610661	-0.765296
C	1.735317	2.552262	-0.397438
C	1.769689	2.215613	2.383197
Ir	-1.719573	1.071858	-0.807574
Ir	1.719526	1.071834	0.807573
N	-0.157135	0.029989	1.584855
N	-2.080706	-0.999429	1.558712
N	2.080705	-0.999475	-1.558669
N	0.157161	0.029984	-1.584869
O	1.821086	3.412718	-1.154585
O	1.731150	2.884449	3.305070
O	-1.731395	2.884551	-3.304994
O	-1.821237	3.412618	1.154725
C	3.182259	-0.036209	1.476791
C	-3.181815	-0.036676	-1.477109
O	4.134293	-0.478010	1.937792
O	-4.133572	-0.478862	-1.938318

$3_{H\text{-}T}^{+1}$

Zero-point correction= 0.818363 (Hartree/Particle)
Thermal correction to Energy= 0.864221
Thermal correction to Enthalpy= 0.865166
Thermal correction to Gibbs Free Energy= 0.738645
Sum of electronic and zero-point Energies= -1981.958209
Sum of electronic and thermal Energies= -1981.912351
Sum of electronic and thermal Enthalpies= -1981.911407
Sum of electronic and thermal Free Energies= -1982.037927

C	1.302356	-0.649068	-0.889871
C	0.299003	-1.561891	-2.650370
H	-0.502915	-1.775705	-3.334642
C	1.572696	-2.034659	-2.634294
H	2.087176	-2.729317	-3.273669
C	3.551553	-1.704307	-1.135518
C	4.593674	-1.151528	-1.891606
C	5.899863	-1.376784	-1.466654
H	6.717071	-0.952973	-2.039484
C	6.184011	-2.128497	-0.325472
C	5.119450	-2.669738	0.391354
H	5.323364	-3.272242	1.269563
C	3.793101	-2.475201	0.005102
C	4.314858	-0.315149	-3.114021

H	5.205330	0.232740	-3.419502
H	3.513581	0.404353	-2.931622
H	3.998010	-0.932399	-3.957758
C	7.606704	-2.328188	0.127424
H	8.282799	-2.437453	-0.721816
H	7.705745	-3.212887	0.757057
H	7.951167	-1.468572	0.709634
C	2.670558	-3.080713	0.806405
H	3.024737	-3.939007	1.376889
H	1.845426	-3.403839	0.171464
H	2.257622	-2.357362	1.513134
C	2.352695	2.280096	-0.054639
H	2.102053	2.302327	-1.109914
C	3.314305	1.339651	0.333824
H	3.768254	0.744821	-0.447040
C	4.121029	1.365157	1.614490
H	4.993794	2.015782	1.485329
H	4.515910	0.357389	1.763111
C	3.323272	1.791005	2.867447
H	3.349066	2.873670	2.987444
H	3.811821	1.378271	3.750875
C	1.876624	1.321238	2.847805
H	1.634399	0.510099	3.528000
C	0.812273	2.064645	2.360023
H	-0.179780	1.782919	2.697694
C	0.909242	3.429771	1.723567
H	0.997262	4.197972	2.500325
H	-0.034305	3.617420	1.213492
C	2.063606	3.565547	0.710560
H	1.818833	4.354494	-0.002168
H	2.974098	3.893733	1.211552
C	-1.302333	-0.651107	0.886713
C	-1.572644	-2.041660	2.627277
H	-2.087263	-2.737832	3.264884
C	-0.298912	-1.569096	2.644581
H	0.503041	-1.784984	3.328161
C	-3.552677	-1.704811	1.132038
C	-4.591880	-1.149534	1.890138
C	-5.899300	-1.364920	1.463330
H	-6.713940	-0.933859	2.034380
C	-6.187326	-2.111739	0.320014
C	-5.125149	-2.651503	-0.401759
H	-5.331835	-3.242009	-1.287471
C	-3.798083	-2.467051	-0.013738
C	-4.308345	-0.314335	3.112265
H	-5.197257	0.234264	3.420972
H	-3.506771	0.404409	2.927989
H	-3.989530	-0.932594	3.954496
C	-7.613081	-2.356479	-0.099051
H	-8.251198	-1.504679	0.140930
H	-8.021597	-3.227119	0.421659
H	-7.688945	-2.549692	-1.169742
C	-2.678575	-3.068499	-0.822357
H	-2.267999	-2.341757	-1.527027
H	-3.034961	-3.923883	-1.395851
H	-1.851231	-3.394954	-0.192080
C	-2.351561	2.281122	0.059241
H	-2.101678	2.300359	1.114771
C	-3.313589	1.342445	-0.332428
H	-3.768466	0.745831	0.446518
C	-4.119649	1.372003	-1.613426
H	-4.992263	2.022486	-1.482708
H	-4.515086	0.364966	-1.765028
C	-3.321128	1.801081	-2.864738
H	-3.809618	1.391254	-3.749549
H	-3.346118	2.884117	-2.981628
C	-1.874854	1.330294	-2.845893
H	-1.632827	0.520972	-3.528323
C	-0.810232	2.071569	-2.355547
H	0.181772	1.790166	-2.693648
C	-0.906651	3.434930	-1.715250
H	-0.994115	4.205314	-2.489913
H	0.036890	3.620740	-1.204490
C	-2.061242	3.568543	-0.702194
H	-1.816173	4.355283	0.012874
H	-2.971352	3.898723	-1.202568
Ir	1.347440	0.502341	0.802999
Ir	-1.346854	0.505118	-0.803034
N	0.149648	-0.722775	-1.563294
N	2.197270	-1.453174	-1.537788
N	-2.197233	-1.457106	1.532434
N	-0.149556	-0.726945	1.559864

$7_{H\text{-}T}$

Zero-point correction= 0.668043 (Hartree/Particle)
Thermal correction to Energy= 0.710442
Thermal correction to Enthalpy= 0.711386
Thermal correction to Gibbs Free Energy= 0.591260
Sum of electronic and zero-point Energies= -1672.475794
Sum of electronic and thermal Energies= -1672.433395
Sum of electronic and thermal Enthalpies= -1672.432450
Sum of electronic and thermal Free Energies= -1672.552576

C	1.383158	0.228149	0.752759
C	0.525299	1.111435	2.606674
H	-0.219485	1.311169	3.356580
C	1.780736	1.614249	2.487088
H	2.332235	2.321997	3.079711
C	3.616230	1.361123	0.826721
C	4.739865	0.823437	1.465394
C	5.998409	1.134823	0.956118
H	6.876340	0.718406	1.438068
C	6.155290	1.954472	-0.160667
C	5.013697	2.477512	-0.763567
H	5.118877	3.124491	-1.627857
C	3.733333	2.198023	-0.287157
C	4.592942	-0.095112	2.650118
H	5.547162	-0.557167	2.902190
H	3.871238	-0.887208	2.443421
H	4.231131	0.438869	3.531585
C	7.525321	2.238942	-0.721508
H	8.285386	2.239582	0.061680
H	7.556896	3.205678	-1.226797
H	7.809866	1.476960	-1.453331
C	2.518473	2.766479	-0.968854
H	2.786684	3.619666	-1.592616
H	1.763388	3.082477	-0.247805
H	2.048948	2.008941	-1.599239
C	2.468248	-2.455705	0.083589
H	2.409762	-2.287040	1.152726
C	3.360165	-1.708275	-0.702669
H	4.016749	-0.973715	-0.257086
C	1.508700	-1.545811	-3.097555
H	1.030564	-0.787299	-3.705485
C	0.739223	-2.487323	-2.422588
H	-0.342442	-2.457735	-2.476421
C	-1.384143	0.227203	-0.751150
C	-1.783852	1.618900	-2.480528
H	-2.336535	2.327864	-3.070591
C	-0.527673	1.118443	-2.601956
H	0.216794	1.322003	-3.351150
C	-3.619147	1.357854	-0.821947
C	-4.741397	0.817103	-1.460800
C	-6.000555	1.120771	-0.948579
H	-6.876979	0.696652	-1.426535
C	-6.159684	1.938882	0.169072
C	-5.019286	2.459468	0.776157
H	-5.125682	3.095810	1.648222
C	-3.738238	2.187455	0.297083
C	-4.591229	-0.102748	-2.644092
H	-5.545426	-0.562468	-2.900460
H	-3.872555	-0.896599	-2.433477
H	-4.223743	0.429755	-3.524052
C	-7.534057	2.275231	0.689005
H	-8.239625	1.463260	0.504495
H	-7.927759	3.169511	0.196552
H	-7.516778	2.472951	1.762039
C	-2.525068	2.750119	0.986557
H	-2.061973	1.989551	1.618119
H	-2.793598	3.603166	1.610406
H	-1.764712	3.064538	0.270473
C	-2.461947	-2.462371	-0.088972
H	-2.402259	-2.292000	-1.157779
C	-3.356244	-1.717788	0.697290
H	-4.013735	-0.983789	0.252164
C	-1.505868	-1.559537	3.093486
H	-1.031923	-0.800852	3.704504
C	-0.731374	-2.495244	2.416233
H	0.350068	-2.460717	2.471382
Ir	1.354623	-0.925502	-0.964659
Ir	-1.352511	-0.931565	0.962662
N	0.292336	0.269441	1.538401
N	2.315935	1.050512	1.336600
N	-2.318143	1.050362	-1.331969
N	-0.293407	0.272972	-1.536697
H	3.741676	-2.136013	-1.623628
H	2.166731	-3.451999	-0.218741
H	2.534870	-1.759596	-3.369438
H	1.154998	-3.452434	-2.157691
H	-3.738193	-2.147491	1.617109
H	-2.158939	-3.458540	0.212255
H	-1.142282	-3.461434	2.147761
H	-2.531294	-1.779019	3.363638

Rh-$3_{H\text{-}H}$

Zero-point correction= 0.815904 (Hartree/Particle)
Thermal correction to Energy= 0.861632
Thermal correction to Enthalpy= 0.862576
Thermal correction to Gibbs Free Energy= 0.737580
Sum of electronic and zero-point Energies= -1994.505452
Sum of electronic and thermal Energies= -1994.459724
Sum of electronic and thermal Enthalpies= -1994.458780
Sum of electronic and thermal Free Energies= -1994.583776

C	-0.160678	1.066035	-0.942193
C	-1.375210	1.627066	-2.723725
H	-2.197271	1.575255	-3.416532
C	-0.249721	2.381454	-2.784578
H	0.094070	3.107185	-3.500120
C	1.585093	2.855087	-1.186961
C	1.288263	4.138913	-0.705218
C	2.331268	4.919519	-0.209887
H	2.106168	5.907771	0.176699
C	3.643798	4.455378	-0.173383
C	3.900243	3.170816	-0.646746
H	4.913266	2.784599	-0.613223
C	2.891534	2.356015	-1.158084
C	-0.124169	4.665420	-0.677041
H	-0.424386	5.057210	-1.651809
H	-0.217320	5.471282	0.051358
H	-0.838026	3.882324	-0.421288
C	4.758752	5.327461	0.344205
H	4.406192	6.004896	1.124038
H	5.176072	5.942256	-0.459062
H	5.573970	4.730088	0.755921
C	3.195634	0.960038	-1.612748
H	2.775686	0.770292	-2.599826
H	2.749868	0.232130	-0.930218
H	4.270558	0.782824	-1.649544
C	-0.125258	1.741141	2.048801
H	-0.647039	2.432480	1.396840
C	1.254527	1.700664	1.957799
H	1.724038	2.341148	1.227307
C	2.174537	1.187368	3.048814
H	3.096817	1.772615	3.033797
H	1.723651	1.360859	4.027420
C	2.524506	-0.302871	2.877508
H	2.892625	-0.716763	3.826666
H	3.349768	-0.391856	2.166309
C	1.387169	-1.140039	2.343532
H	1.715052	-2.044440	1.855537
C	0.050794	-1.055188	2.702198
H	-0.584177	-1.894901	2.441330
C	-0.509884	-0.125294	3.766239
H	-1.369848	-0.607052	4.237805
H	0.228597	0.008138	4.558579
C	-0.951815	1.248017	3.212797
H	-0.950479	1.992761	4.020419
H	-1.983327	1.175250	2.866786
C	-0.178963	-1.712712	-0.321483
C	-1.593402	-3.153894	-1.276198
H	-2.530646	-3.505618	-1.670923
C	-0.393045	-3.782491	-1.228703
H	-0.066949	-4.746660	-1.576717
C	1.902060	-3.069809	-0.637813
C	2.530183	-3.826656	0.353213
C	3.922012	-3.934684	0.313061
H	4.424477	-4.501396	1.089926
C	4.675805	-3.340150	-0.694382
C	4.005703	-2.653911	-1.708811
H	4.575059	-2.220660	-2.524258
C	2.621438	-2.518411	-1.707868
C	1.743430	-4.552827	1.414972
H	2.293124	-4.583718	2.356737
H	1.556812	-5.585806	1.106920
H	0.775396	-4.088588	1.594334
C	6.181200	-3.421236	-0.688247
H	6.580730	-3.487422	-1.702080
H	6.533515	-4.288115	-0.126951
H	6.615534	-2.530564	-0.224067
C	1.892725	-1.859014	-2.848785
H	2.593729	-1.459161	-3.580580
H	1.246668	-1.052747	-2.501909
H	1.247321	-2.582690	-3.352851
C	-3.875798	-1.346808	0.944818
H	-3.218779	-2.141283	1.285085
C	-4.538622	-1.545557	-0.270016
H	-4.355633	-2.481165	-0.787946
C	-5.829970	-0.833799	-0.651025
H	-6.450573	-1.508729	-1.244264
H	-6.404170	-0.614536	0.251075
C	-5.576614	0.458493	-1.454294
H	-6.432941	1.139790	-1.359935
H	-5.503300	0.205426	-2.514726
C	-4.291443	1.159827	-1.069988
H	-3.884423	1.799252	-1.846081
C	-3.841504	1.388818	0.233108
H	-3.081125	2.153507	0.360849
C	-4.627146	1.034521	1.488632
H	-5.696043	1.121246	1.283958
H	-4.410070	1.772335	2.264090
C	-4.297364	-0.376523	2.025465

H	-3.469571	-0.305525	2.732169
H	-5.149750	-0.775727	2.590705
N	-1.315046	0.837696	-1.591553
N	0.502903	2.045354	-1.664371
N	-1.446502	-1.894560	-0.730952
N	0.483966	-2.886858	-0.626661
Rh	0.333944	0.002140	0.762871
Rh	-2.788845	-0.310146	-0.592090

Rh-3$_{H-H}$

Zero-point correction= 0.815904 (Hartree/Particle)
Thermal correction to Energy= 0.861632
Thermal correction to Enthalpy= 0.862576
Thermal correction to Gibbs Free Energy= 0.737580
Sum of electronic and zero-point Energies= -1994.505452
Sum of electronic and thermal Energies= -1994.459724
Sum of electronic and thermal Enthalpies= -1994.458780
Sum of electronic and thermal Free Energies= -1994.583776

C	-0.160678	1.066035	-0.942193
C	-1.375210	1.627066	-2.723725
H	-2.197271	1.575255	-3.416532
C	-0.249721	2.381454	-2.784578
H	0.094070	3.107185	-3.500120
C	1.585093	2.855087	-1.186961
C	1.288263	4.138913	-0.705218
C	2.331268	4.919519	-0.209887
H	2.106168	5.907771	0.176699
C	3.643798	4.455378	-0.173383
C	3.900243	3.170816	-0.646746
H	4.913266	2.784599	-0.613223
C	2.891534	2.356015	-1.158084
C	-0.124169	4.665420	-0.677041
H	-0.424386	5.057210	-1.651809
H	-0.217320	5.471282	0.051358
H	-0.838026	3.882324	-0.421288
C	4.758752	5.327461	0.344205
H	4.406192	6.004896	1.124038
H	5.176072	5.942256	-0.459062
H	5.573970	4.730088	0.755921
C	3.195634	0.960038	-1.612748
H	2.775686	0.770292	-2.599826
H	2.749868	0.232130	-0.930218
H	4.270558	0.782824	-1.649544
C	-0.125258	1.741141	2.048801
H	-0.647039	2.432480	1.396840
C	1.254527	1.700664	1.957799
H	1.724038	2.341148	1.227307
C	2.174537	1.187368	3.048814
H	3.096817	1.772615	3.033797
H	1.723651	1.360859	4.027420
C	2.524506	-0.302871	2.877508
H	2.892625	-0.716763	3.826666
H	3.349768	-0.391856	2.166309
C	1.387169	-1.140039	2.343532
H	1.715052	-2.044440	1.855537
C	0.050794	-1.055188	2.702198
H	-0.584177	-1.894901	2.441330
C	-0.509884	-0.125294	3.766239
H	-1.369848	-0.607052	4.237805
H	0.228597	0.008138	4.558579
C	-0.951815	1.248017	3.212797
H	-0.950479	1.992761	4.020419
H	-1.983327	1.175250	2.866786
C	-0.178963	-1.712712	-0.321483
C	-1.593402	-3.153894	-1.276198
H	-2.530646	-3.505618	-1.670923
C	-0.393045	-3.782491	-1.228703
H	-0.066949	-4.746660	-1.576717
C	1.902060	-3.069809	-0.637813
C	2.530183	-3.826656	0.353213
C	3.922012	-3.934684	0.313061
H	4.424477	-4.501396	1.089926
C	4.675805	-3.340150	-0.694382
C	4.005703	-2.653911	-1.708811
H	4.575059	-2.220660	-2.524258
C	2.621438	-2.518411	-1.707868
C	1.743430	-4.552827	1.414972
H	2.293124	-4.583718	2.356737
H	1.556812	-5.585806	1.106920
H	0.775396	-4.088588	1.594334
C	6.181200	-3.421236	-0.688247
H	6.580730	-3.487422	-1.702080
H	6.533515	-4.288115	-0.126951
H	6.615534	-2.530564	-0.224067
C	1.892725	-1.859014	-2.848785
H	2.593729	-1.459161	-3.580580
H	1.246668	-1.052747	-2.501909
H	1.247321	-2.582690	-3.352851
C	-3.875798	-1.346808	0.944818
H	-3.218779	-2.141283	1.285085
C	-4.538622	-1.545557	-0.270016
H	-4.355633	-2.481165	-0.787946
C	-5.829970	-0.833799	-0.651025
H	-6.450573	-1.508729	-1.244264

H	-6.404170	-0.614536	0.251075
C	-5.576614	0.458493	-1.454294
H	-6.432941	1.139790	-1.359935
H	-5.503300	0.205426	-2.514726
C	-4.291443	1.159827	-1.069988
H	-3.884423	1.799252	-1.846081
C	-3.841504	1.388818	0.233108
H	-3.081125	2.153507	0.360849
C	-4.627146	1.034521	1.488632
H	-5.696043	1.121246	1.283958
H	-4.410070	1.772335	2.264090
C	-4.297364	-0.376523	2.025465
H	-3.469571	-0.305525	2.732169
H	-5.149750	-0.775727	2.590705
N	-1.315046	0.837696	-1.591553
N	0.502903	2.045354	-1.664371
N	-1.446502	-1.894560	-0.730952
N	0.483966	-2.886858	-0.626661
Rh	0.333944	0.002140	0.762871
Rh	-2.788845	-0.310146	-0.592090

$Rh\text{-}3_{H\text{-}T}$

Zero-point correction= 0.814880 (Hartree/Particle)
Thermal correction to Energy= 0.861192
Thermal correction to Enthalpy= 0.862136
Thermal correction to Gibbs Free Energy= 0.734774
Sum of electronic and zero-point Energies= -1994.513303
Sum of electronic and thermal Energies= -1994.466992
Sum of electronic and thermal Enthalpies= -1994.466048
Sum of electronic and thermal Free Energies= -1994.593410

C	1.332901	-0.495156	-0.849500
C	1.593621	-1.895272	-2.606486
H	2.100039	-2.597794	-3.244240
C	0.327574	-1.403027	-2.614110
H	-0.478042	-1.607480	-3.298269
C	3.559891	-1.617534	-1.109976
C	3.780784	-2.419103	0.014438
C	5.100019	-2.669172	0.392288
H	5.283855	-3.284091	1.266947
C	6.181390	-2.155485	-0.319384
C	5.922241	-1.365815	-1.439138
H	6.751935	-0.945353	-1.997250
C	4.621975	-1.083343	-1.850564
C	2.633903	-2.975222	0.813455
H	2.203325	-2.200819	1.451156
H	2.963803	-3.802139	1.443294
H	1.827412	-3.324550	0.167538
C	7.597603	-2.468434	0.091750
H	7.965089	-3.359707	-0.425803
H	7.668929	-2.659680	1.163754
H	8.273897	-1.647167	-0.152318
C	4.362046	-0.177604	-3.026012
H	3.915131	-0.719546	-3.862079
H	5.287066	0.281829	-3.374054
H	3.660878	0.615445	-2.756978
C	-1.336155	-0.485122	0.860398
C	-1.597654	-1.861131	2.636105
H	-2.104187	-2.555033	3.283164
C	-0.331587	-1.368721	2.637541
H	0.473767	-1.563896	3.324693
C	-3.559762	-1.609696	1.128036
C	-3.768373	-2.440217	0.022262
C	-5.083307	-2.704572	-0.360012
H	-5.258293	-3.346844	-1.216626
C	-6.172228	-2.174323	0.328073
C	-5.925305	-1.363192	1.434937
H	-6.761171	-0.940505	1.982012
C	-4.629254	-1.066156	1.850141
C	-2.611399	-3.019870	-0.744972
H	-2.158555	-2.260837	-1.385376
H	-2.937021	-3.853124	-1.368573
H	-1.822876	-3.368094	-0.076360
C	-7.582505	-2.441693	-0.132923
H	-7.915776	-1.667401	-0.830616
H	-8.282159	-2.449641	0.704681
H	-7.658673	-3.400684	-0.648206
C	-4.381953	-0.142552	3.014358
H	-3.940857	-0.671087	3.862048
H	-5.311101	0.319453	3.347701
H	-3.680651	0.648426	2.739744
C	2.552988	2.289119	0.036427
H	2.358983	2.272109	-1.031356
C	3.447057	1.331437	0.525684
H	3.911559	0.673180	-0.194974
C	4.190864	1.387729	1.841405
H	5.091814	2.009244	1.745495
H	4.542257	0.374403	2.051532
C	3.332365	1.877857	3.026362
H	3.758432	1.495871	3.956894
H	3.375550	2.965603	3.101238
C	1.884345	1.432970	2.922697
H	1.586713	0.630865	3.590199
C	0.873090	2.184253	2.349611
H	-0.147306	1.924505	2.611246

C	1.029809	3.529109	1.678786
H	1.085591	4.329253	2.428892
H	0.117534	3.708784	1.107660
C	2.241924	3.611644	0.726092
H	2.043508	4.371367	-0.033557
H	3.125604	3.955144	1.267239
C	-0.875621	2.153850	-2.372020
H	0.143929	1.889038	-2.631969
C	-1.889188	1.397546	-2.934331
H	-1.594146	0.586473	-3.592109
C	-3.336590	1.843775	-3.041221
H	-3.764692	1.451449	-3.966502
H	-3.377924	2.930627	-3.128971
C	-4.194270	1.369688	-1.849119
H	-5.093707	1.994408	-1.759551
H	-4.548050	0.354497	-2.046389
C	-3.448342	1.328149	-0.534069
H	-3.912899	0.679987	0.195589
C	-2.551775	2.290021	-0.057726
H	-2.356264	2.285196	1.009896
C	-2.238769	3.603552	-0.763410
H	-2.037180	4.371636	-0.013076
H	-3.122514	3.943036	-1.306995
C	-1.028365	3.506956	-1.717019
H	-1.083422	4.298386	-2.476346
H	-0.114852	3.691120	-1.149221
N	2.220074	-1.319407	-1.507528
N	0.180031	-0.557534	-1.534122
N	-2.223582	-1.300200	1.529152
N	-0.183575	-0.538006	1.546219
Rh	1.434923	0.636313	0.877832
Rh	-1.438231	0.625214	-0.880587

Rh-4$_{H-H}$

Zero-point correction= 0.483955 (Hartree/Particle)
Thermal correction to Energy= 0.523639
Thermal correction to Enthalpy= 0.524583
Thermal correction to Gibbs Free Energy= 0.408273
Sum of electronic and zero-point Energies= -1824.048328
Sum of electronic and thermal Energies= -1824.008644
Sum of electronic and thermal Enthalpies= -1824.007700
Sum of electronic and thermal Free Energies= -1824.124010

C	-0.693293	1.237153	-0.472209
C	-2.128194	2.029327	-1.979817
H	-3.037183	2.067194	-2.554375
C	-1.031393	2.823891	-2.042893
H	-0.791336	3.672134	-2.658676
C	1.043772	3.036437	-0.698338
C	0.897610	4.210565	0.052175
C	2.048291	4.897474	0.430091
H	1.946936	5.801308	1.020632
C	3.320512	4.443533	0.089112
C	3.426169	3.269985	-0.653632
H	4.408570	2.900463	-0.927229
C	2.303687	2.549478	-1.058117
C	-0.460625	4.701181	0.481267
H	-1.010539	5.146375	-0.351094
H	-0.367065	5.452577	1.264727
H	-1.073425	3.882790	0.863201
C	4.552155	5.179485	0.550126
H	4.362451	6.248928	0.655223
H	5.380600	5.047202	-0.147838
H	4.881335	4.807458	1.524933
C	2.451537	1.268158	-1.825706
H	1.725535	1.200326	-2.636049
H	2.278097	0.413767	-1.167659
H	3.453622	1.176136	-2.245446
C	0.697704	1.522425	2.013366
C	0.975629	-1.157583	2.269405
C	-0.588920	-1.618360	-0.129023
C	-2.074147	-2.951624	-1.124116
H	-3.038491	-3.255583	-1.491902
C	-0.880422	-3.590765	-1.196132
H	-0.589939	-4.530752	-1.630181
C	1.441912	-3.019638	-0.516483
C	1.968475	-3.791117	0.521440
C	3.351158	-3.976863	0.559635
H	3.776499	-4.556362	1.371868
C	4.192538	-3.438096	-0.409501
C	3.620727	-2.725792	-1.464875
H	4.258853	-2.330273	-2.247743
C	2.247737	-2.516161	-1.545945
C	1.084906	-4.451808	1.547564
H	1.590907	-4.518574	2.510277
H	0.832975	-5.467477	1.228814
H	0.148919	-3.915520	1.690742
C	5.687707	-3.602010	-0.313766
H	6.148430	-3.648556	-1.302170
H	5.956737	-4.508762	0.230187
H	6.135206	-2.756185	0.216710
C	1.632975	-1.833259	-2.739202
H	2.390865	-1.316321	-3.326555
H	0.869301	-1.113960	-2.447516
H	1.148218	-2.568917	-3.386905

	C	-4.490350	-1.502327	0.586040
	C	-4.536475	1.154123	0.031728
	N	-1.910908	1.064363	-1.014939
	N	-0.145440	2.335820	-1.094073
	N	-1.880747	-1.745935	-0.477704
	N	0.034686	-2.760480	-0.570117
	Rh	0.184813	0.004052	0.982041
	Rh	-3.306940	-0.255385	-0.172140
	O	-5.267604	2.023520	0.147467
	O	-5.193467	-2.275541	1.046136
	O	1.459197	-1.793134	3.087255
	O	0.959155	2.399824	2.697035

Rh-4_{H-T}

Zero-point correction= 0.482697 (Hartree/Particle)
Thermal correction to Energy= 0.523199
Thermal correction to Enthalpy= 0.524143
Thermal correction to Gibbs Free Energy= 0.403891
Sum of electronic and zero-point Energies= -1824.054000
Sum of electronic and thermal Energies= -1824.013499
Sum of electronic and thermal Enthalpies= -1824.012554
Sum of electronic and thermal Free Energies= -1824.132806

C	1.386773	-0.748821	-0.035877
C	0.551075	-2.632258	0.786880
H	-0.184576	-3.400539	0.950303
C	1.810770	-2.524599	1.282604
H	2.379173	-3.147048	1.950255
C	3.626808	-0.827158	1.058195
C	4.743798	-1.374497	0.419797
C	5.994987	-0.837556	0.716461
H	6.869535	-1.244160	0.220602
C	6.147918	0.210842	1.621508
C	5.011195	0.717979	2.249451
H	5.115763	1.526731	2.964594
C	3.739237	0.214918	1.985410
C	4.601664	-2.504896	-0.565272
H	5.516641	-2.631037	-1.143223
H	3.780427	-2.327089	-1.260048
H	4.389785	-3.448814	-0.056445
C	7.506209	0.807049	1.888996
H	8.299447	0.068864	1.760503
H	7.574147	1.207124	2.902083
H	7.708939	1.630065	1.197218
C	2.524079	0.784710	2.667688
H	2.811001	1.398324	3.521747
H	1.853387	-0.003257	3.014827
H	1.947949	1.404235	1.977314
C	3.079860	0.563906	-1.869976
C	1.459693	2.796411	-1.901812
C	-1.386733	0.748742	-0.035961
C	-1.810716	2.524806	1.282144
H	-2.379112	3.147397	1.949667
C	-0.551001	2.632322	0.786437
H	0.184683	3.400595	0.949749
C	-3.626800	0.827382	1.058027
C	-4.743751	1.374701	0.419520
C	-5.994971	0.837899	0.716252
H	-6.869488	1.244484	0.220321
C	-6.147988	-0.210369	1.621455
C	-5.011317	-0.717502	2.249465
H	-5.115935	-1.526165	2.964701
C	-3.739310	-0.214559	1.985371
C	-4.601518	2.504923	-0.565739
H	-5.516424	2.630928	-1.143832
H	-3.780182	2.327011	-1.260369
H	-4.389736	3.448943	-0.057061
C	-7.506357	-0.806373	1.889004
H	-8.299437	-0.067910	1.761109
H	-7.574132	-1.206933	2.901910
H	-7.709496	-1.628979	1.196863
C	-2.524210	-0.784359	2.667746
H	-1.948165	-1.404093	1.977486
H	-2.811194	-1.397766	3.521932
H	-1.853418	0.003598	3.014716
C	-3.079906	-0.564315	-1.869732
C	-1.459646	-2.796822	-1.901252
Rh	1.490933	1.078821	-1.036042
Rh	-1.490928	-1.079071	-1.035801
N	0.301265	-1.535681	-0.016195
N	2.327396	-1.350940	0.758052
N	-2.327353	1.351045	0.757832
N	-0.301196	1.535573	-0.016400
O	-4.044554	-0.245888	-2.395785
O	-1.423672	-3.820594	-2.402935
O	1.423801	3.820080	-2.403711
O	4.044466	0.245369	-2.396038

Rh-5

Zero-point correction= 0.695978 (Hartree/Particle)
Thermal correction to Energy= 0.747157
Thermal correction to Enthalpy= 0.748101
Thermal correction to Gibbs Free Energy= 0.607682
Sum of electronic and zero-point Energies= -2519.488990
Sum of electronic and thermal Energies= -2519.437810
Sum of electronic and thermal Enthalpies= -2519.436866
Sum of electronic and thermal Free Energies= -2519.577285

C	-1.393363	-0.773097	-0.495818
C	-0.512369	-2.589286	-1.414176
H	0.252784	-3.310326	-1.645811
C	-1.795732	-2.521561	-1.860438
H	-2.359802	-3.139867	-2.536060
C	-3.667004	-0.898017	-1.495067
C	-4.733539	-1.486459	-0.808755
C	-6.013931	-0.985470	-1.032994
H	-6.850460	-1.428714	-0.503612
C	-6.243058	0.075903	-1.906958
C	-5.154553	0.634495	-2.574286
H	-5.317826	1.458152	-3.261071
C	-3.856356	0.164396	-2.384373
C	-4.498530	-2.607839	0.168672
H	-5.436900	-2.936352	0.615032
H	-3.836317	-2.284125	0.973383
H	-4.025929	-3.467714	-0.310381
C	-7.632776	0.629277	-2.096068
H	-8.388143	-0.155715	-2.026724
H	-7.739497	1.118602	-3.065656
H	-7.861898	1.371882	-1.325809
C	-2.687843	0.790382	-3.096330
H	-3.026043	1.477788	-3.872141
H	-2.049137	0.033352	-3.555550
H	-2.061218	1.339428	-2.390407
C	-2.897362	0.466519	1.556881
C	1.394314	0.772872	-0.491552
C	1.798556	2.523641	-1.852721
H	2.363687	3.143137	-2.526361
C	0.514562	2.590611	-1.408171
H	-0.250337	3.311943	-1.639735
C	3.669969	0.901024	-1.487108
C	4.733542	1.486069	-0.793998
C	6.014664	0.983341	-1.012648
H	6.848214	1.419851	-0.473232
C	6.247100	-0.073548	-1.890463
C	5.160076	-0.633462	-2.559914
H	5.324820	-1.461584	-3.241112
C	3.861953	-0.162221	-2.375540
C	4.494610	2.601622	0.189143
H	5.431204	2.927740	0.640950
H	3.829105	2.273787	0.989376
H	4.024092	3.464099	-0.287302
C	7.645940	-0.581364	-2.132923
H	8.282028	-0.429215	-1.259258
H	8.110970	-0.056229	-2.972888
H	7.647173	-1.646191	-2.372628
C	2.694957	-0.791608	-3.086985
H	2.069817	-1.342208	-2.380964
H	3.034868	-1.478038	-3.862917
H	2.054244	-0.036227	-3.546092
C	2.892741	-0.471264	1.563921
Rh	-1.408557	0.963161	0.613714
Rh	1.406993	-0.965632	0.614692
N	-0.279687	-1.515337	-0.577451
N	-2.340762	-1.387109	-1.273802
N	2.342734	1.388143	-1.267337
N	0.280759	1.515270	-0.573568
O	3.826149	-0.164571	2.171423
O	-3.833312	0.158091	2.159639
P	0.947427	-2.839688	1.945148
P	-0.951313	2.833897	1.949637
C	-0.858180	-2.977014	2.310080
H	-1.064501	-3.808602	2.986664
H	-1.202619	-2.041534	2.749826
H	-1.392288	-3.121193	1.371783
C	1.703894	-2.894464	3.630341
H	1.368899	-3.771171	4.187916
H	2.790194	-2.913596	3.539446
H	1.424289	-1.990896	4.172192
C	1.337479	-4.530191	1.298005
H	2.409543	-4.610065	1.118026
H	1.027040	-5.304455	2.003045
H	0.814624	-4.676698	0.353264
C	0.853570	2.969486	2.318694
H	1.058809	3.799099	2.998022
H	1.196784	2.032640	2.756482
H	1.389608	3.116071	1.381885
C	-1.339126	4.526291	1.306040
H	-2.410831	4.607352	1.124456
H	-1.029349	5.298637	2.013477
H	-0.814601	4.674737	0.362525
C	-1.711308	2.884601	3.633347
H	-1.377050	3.759584	4.194063
H	-2.797397	2.904555	3.540171
H	-1.433334	1.979382	4.173278

Rh-6

Zero-point correction= 0.736760 (Hartree/Particle)
Thermal correction to Energy= 0.791090
Thermal correction to Enthalpy= 0.792034
Thermal correction to Gibbs Free Energy= 0.647620
Sum of electronic and zero-point Energies= -2570.834379
Sum of electronic and thermal Energies= -2570.780049
Sum of electronic and thermal Enthalpies= -2570.779104
Sum of electronic and thermal Free Energies= -2570.923519

C	1.607093	-0.549908	-0.717091
C	0.435920	-1.573278	-2.292391
H	-0.435165	-1.778489	-2.890951
C	1.671962	-2.143411	-2.289867
H	2.101841	-2.939542	-2.871619
C	3.726906	-1.864257	-0.896799
C	4.828941	-1.230711	-1.471304
C	6.100133	-1.610598	-1.037113
H	6.967925	-1.128268	-1.473721
C	6.280458	-2.590862	-0.065165
C	5.151855	-3.210514	0.474213
H	5.277806	-3.980260	1.227984
C	3.865920	-2.863649	0.074084
C	4.660190	-0.151579	-2.508959
H	5.571197	-0.034004	-3.096300
H	4.444241	0.810657	-2.039270
H	3.836612	-0.371427	-3.188445
C	7.659430	-2.964769	0.415456
H	8.429616	-2.639018	-0.284969
H	7.754832	-4.044497	0.547204
H	7.872390	-2.498738	1.381941
C	2.650909	-3.518189	0.674766
H	2.935205	-4.248918	1.431680
H	2.056042	-4.026660	-0.087262
H	1.994885	-2.778204	1.135942
C	2.683438	2.016903	-0.086168
C	-0.914438	-0.445182	1.145179
C	-1.040518	-1.878887	2.868228
H	-1.505863	-2.592613	3.524642
C	0.224915	-1.382571	2.809526
H	1.074416	-1.586051	3.438016
C	-3.099989	-1.636703	1.500161
C	-4.153406	-0.880019	2.010992
C	-5.452282	-1.262434	1.676611
H	-6.283487	-0.678554	2.057051
C	-5.705564	-2.364512	0.866068
C	-4.624127	-3.105003	0.391309
H	-4.805913	-3.958181	-0.252739
C	-3.312203	-2.755453	0.687835
C	-3.914047	0.334486	2.868392
H	-4.782823	0.543165	3.493798
H	-3.737975	1.214902	2.246939
H	-3.046563	0.209797	3.516881
C	-7.112886	-2.727067	0.467840
H	-7.845824	-2.316229	1.164119
H	-7.250082	-3.809578	0.427441
H	-7.340919	-2.330592	-0.525782
C	-2.150763	-3.503783	0.093706
H	-1.618629	-2.855787	-0.606478
H	-2.493302	-4.383506	-0.450593
H	-1.435416	-3.823237	0.854615
C	-1.865039	2.139076	0.341845
Rh	1.594302	0.832288	0.826160
Rh	-0.905374	0.818429	-0.512685
N	0.428076	-0.598256	-1.321567
N	2.405538	-1.491797	-1.299237
N	-1.746830	-1.286405	1.826927
N	0.273736	-0.505756	1.749600
O	-2.464928	2.981259	0.850458
O	3.393802	2.738385	-0.630828
P	-0.341824	2.319665	-2.282186
P	1.068295	2.421669	2.540406
C	1.047142	1.744248	-3.347621
H	1.292761	2.499130	-4.096084
H	1.921013	1.533848	-2.735873
H	0.747262	0.820341	-3.839792
C	0.198436	4.010714	-1.764495
H	0.448082	4.620544	-2.634662
H	-0.615217	4.488531	-1.217814
H	1.064123	3.944521	-1.110766
C	-1.643341	2.733038	-3.517968
H	-2.508160	3.148184	-3.001105
H	-1.262464	3.451645	-4.246801
H	-1.968805	1.819229	-4.010922
C	-0.394527	2.002220	3.575303
H	-0.552306	2.771342	4.332884
H	-1.277712	1.923709	2.948342
H	-0.229396	1.038194	4.053669
C	2.367884	2.717000	3.820455
H	3.279692	3.074698	3.341861
H	2.027083	3.456512	4.547013
H	2.593227	1.783438	4.334686
C	0.709309	4.141350	1.978281
H	0.509102	4.792205	2.831254
H	1.565688	4.527802	1.425400
H	-0.156820	4.131931	1.321514
I	-3.153443	-0.292758	-2.019071
C	3.320541	0.048092	1.850931
H	2.997134	-0.863108	2.351756
H	4.109419	-0.198834	1.146537
H	3.710796	0.749485	2.585315

Rh-7$_{H-T}$

Zero-point correction= 0.666324 (Hartree/Particle)
Thermal correction to Energy= 0.709503
Thermal correction to Enthalpy= 0.710447
Thermal correction to Gibbs Free Energy= 0.588738
Sum of electronic and zero-point Energies= -1684.835320
Sum of electronic and thermal Energies= -1684.792142
Sum of electronic and thermal Enthalpies= -1684.791198
Sum of electronic and thermal Free Energies= -1684.912907

C	-1.394796	-0.048093	0.716389
C	-0.564977	-0.955742	2.565472
H	0.166717	-1.164456	3.326664
C	-1.814944	-1.466492	2.421524
H	-2.372637	-2.183852	2.996837
C	-3.625136	-1.193636	0.737176
C	-4.761331	-0.667818	1.363825
C	-6.010616	-0.971545	0.827023
H	-6.897308	-0.563476	1.300054
C	-6.147398	-1.774291	-0.304361
C	-4.994780	-2.289370	-0.893282
H	-5.084160	-2.925603	-1.767353
C	-3.723548	-2.016269	-0.389649
C	-4.638712	0.227254	2.569147
H	-5.599285	0.679680	2.814735
H	-3.917252	1.026538	2.392344
H	-4.288908	-0.323810	3.444870
C	-7.506790	-2.050959	-0.894384
H	-8.283462	-2.047170	-0.127690
H	-7.532982	-3.017734	-1.400052
H	-7.771493	-1.287866	-1.632515
C	-2.496019	-2.579626	-1.052639
H	-2.754965	-3.413928	-1.705220
H	-1.764741	-2.921179	-0.318812
H	-1.999281	-1.813280	-1.649886
C	-2.496692	2.602048	0.076047
H	-2.443367	2.378110	1.133818
C	-3.370137	1.908149	-0.752927
H	-4.022851	1.139170	-0.364677
C	-1.463441	1.727766	-3.156991
H	-0.965896	0.939376	-3.706241
C	-0.734672	2.667909	-2.468049
H	0.346716	2.628186	-2.442470
C	1.395640	-0.045051	-0.715622
C	1.817644	-1.465702	-2.418474
H	2.376402	-2.183146	-2.992650
C	0.567013	-0.956867	-2.563272
H	-0.164413	-1.167773	-3.324119
C	3.627743	-1.188497	-0.735534
C	4.762620	-0.658029	-1.361114
C	6.012377	-0.954962	-0.821952
H	6.897541	-0.537919	-1.290021
C	6.151338	-1.758702	0.308586
C	4.999892	-2.272812	0.900318
H	5.090340	-2.900264	1.780643
C	3.728011	-2.006266	0.394494
C	4.636726	0.241113	-2.563029
H	5.597458	0.691209	-2.812226
H	3.918684	1.042168	-2.380223
H	4.280602	-0.306287	-3.438443
C	7.515996	-2.088623	0.857595
H	8.225714	-1.280053	0.673961
H	7.917500	-2.990328	0.385350
H	7.479595	-2.270860	1.932931
C	2.502019	-2.565303	1.063917
H	2.012177	-1.797458	1.664978
H	2.761282	-3.401468	1.714016
H	1.764914	-2.902766	0.334157
C	2.490216	2.609940	-0.074858
H	2.435014	2.387810	-1.132935
C	3.366032	1.915732	0.751377
H	4.019174	1.148266	0.360943
C	1.459945	1.734873	3.156234
H	0.967713	0.944079	3.706868
C	0.724932	2.670067	2.467215
H	-0.356209	2.623834	2.442841
Rh	-1.366079	1.092251	-0.998142
Rh	1.363567	1.096547	0.998136
N	-0.318901	-0.090916	1.517438
N	-2.333764	-0.887772	1.270641
N	2.335667	-0.884546	-1.268461
N	0.319814	-0.090632	-1.516659
H	-3.703172	2.357161	-1.681414
H	-2.134753	3.587210	-0.192408
H	-2.493056	1.908838	-3.436702
H	-1.174483	3.617150	-2.187503
H	3.700276	2.363499	1.679999
H	2.127881	3.594321	0.195915
H	1.158657	3.621681	2.185311
H	2.488753	1.922338	3.434672
